# Bridging traditional Chinese medicine theory with advanced MOFs delivery for synergistic drug-resistant infected-chronic wound therapy

**DOI:** 10.1016/j.mtbio.2026.102861

**Published:** 2026-01-30

**Authors:** Chen Chen, Na Zhang, Fructueux Modeste Amona, Xiaolei Han, Qi Tang, Lanxin Geng, Guangfu Liao, Jie Zhang, Tushuai Li

**Affiliations:** a College of Hydraulic Engineering, Jiangsu Vocational Institute of Architectural Technology, Xuzhou, Jiangsu, 221000, China; b School of Food and Biological Engineering, Xuzhou University of Technology, Xuzhou, Jiangsu, 221018, China; c Institute of Cellular and Molecular Biology, School of Life Science, Jiangsu Normal University, Xuzhou, Jiangsu, 221116, China; d School of Biology and Food Engineering, Suzhou University of Technology, Suzhou, Jiangsu, 215500, China; e College of Materials Engineering, Fujian Agriculture and Forestry University, Fuzhou, 350002, China

**Keywords:** Traditional Chinese medicine, Metal-organic frameworks, Synergistic antibacterial therapy, ROS scavenging, Stimuli-responsive drug release, Infected chronic wound healing

## Abstract

Chronic wounds infected with multidrug-resistant bacteria pose a significant challenge to global health, as traditional monotherapies often fail to eradicate pathogens and restore the damaged wound microenvironment. Engineering innovative nanocomposites that integrate synergistic therapeutics and controlled release is a pivotal strategy for advanced wound management. Here, we developed a novel multi-drug metal-organic framework (MOF) nanocomposite, BBR + FA@UiO, by covalently grafting ferulic acid (FA) onto UiO-66-NH_2_ and physically loading berberine (BBR). Drawing from "Jun–Chen–Zuo–[*Shi*]" hierarchy of Traditional Chinese Medicine (TCM), this design used the MOF as a robust porous carrier “[*Shi*]” for the antibacterial drug "Jun" (BBR) and antioxidant drug "Chen/Zuo" (FA). BBR + FA@UiO exhibits potent, synergistic antibacterial and antibiofilm activity against critical clinical pathogens like MRSA and *Pseudomonas aeruginosa, through* a pH/ROS-responsive release mechanism in the infected site. Functionally, BBR + FA@UiO activates the Nrf2/Keap1 pathway in macrophages, thereby mitigating oxidative stress and enhancing mitochondrial function. Consequently, proliferation and migration of Human Umbilical Vein Endothelial Cells (HUVECs) are restored through promoting angiogenesis. *In vivo,* BBR + FA@UiO significantly accelerates healing by eradicating bacteria, enhancing collagen deposition, and improving vascularization, driven by the coordinated activation of the Nrf2/HO-1 antioxidant axis and pro-regenerative Wnt/β-catenin and TGF-β signaling pathways. Demonstrating excellent biocompatibility and no systemic toxicity, this work establishes BBR + FA@UiO as a new therapeutic approach that merges TCM with advanced MOFs for comprehensive wound care.

## Graphical abstract

A multifunctional metal–organic framework (BBR + FA@UiO) simultaneously eliminates reactive oxygen species and eradicates drug-resistant bacteria to promote wound repair. Ferulic acid acts as an antioxidant to restore redox balance, while berberine exerts antibacterial activity. Their synergistic action accelerates MRSA-infected wound healing by reducing inflammation and enhancing vascular regeneration through the TGF-β/β-catenin/Wnt pathway.

**Figure:**
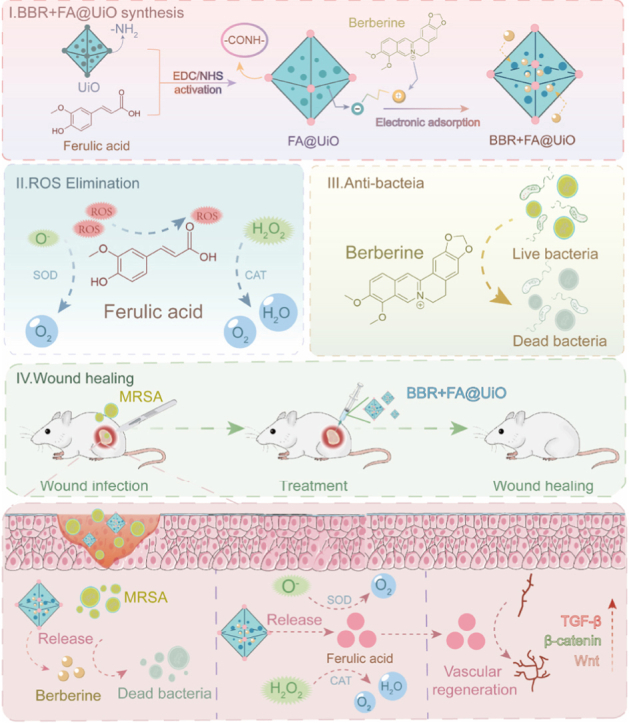

## 1 Introduction

Chronic, non-healing wounds infected with drug-resistant bacteria, such as methicillin-resistant *Staphylococcus aureus* (MRSA) and *Pseudomonas aeruginosa* (PA), present a significant global health threat. These pathogens, classified by the World Health Organization (WHO) as high-priority [1,2], contribute to the persistence and complication of wound healing, affecting approximately 8 million people worldwide [3]. Specifically, conventional monotherapies, such as antibiotics, often fail due to the rise of multidrug-resistant pathogens and their inability to modulate the hostile wound microenvironment. Concurrently, standalone antioxidant or pro-regenerative agents cannot eradicate entrenched infections. This therapeutic gap necessitates the development of novel multifunctional platforms that simultaneously control infection, resolve oxidative damage, and actively promote healing in a coordinated manner.

Traditional Chinese Medicine (TCM) has long recognized the value of plant-based compounds for their multi-target mechanisms and robust safety profiles, making them promising candidates for addressing complex conditions such as chronic wounds. Notably, ingredients such as berberine (BBR) and ferulic acid (FA) can act synergistically to kill drug-resistant bacteria. The TCM theory of "Jun–Chen–Zuo–[*Shi*]", known as the “emperor”–“minister”–“assistant”–[*courier*], offers advanced benefits, as rational combinations of active ingredients provide synergistic therapeutic outcomes [4]. This theory draws on the principle, where "Jun" (emperor) herbs like BBR provide the primary therapeutic action, "Chen/Zuo" (minister/assistant) herbs like FA enhance the primary effect and mitigate the side effects, and "[*Shi*]" (*courier*) herbs guide the compounds to the specific disease site. Additionally, [*Shi*] can help to harmonize drugs in a formula to eradicate pathogens. This framework aligns with modern scientific research supporting the use of TCM compounds for multi-target therapeutic strategies.

WHO recognizes that TCM's use of multi-targeting, multi-component drugs provides a rationale for conducting clinical trials worldwide [5]. BBR, a significant isoquinoline alkaloid, exhibits strong broad-spectrum antimicrobial and anti-inflammatory properties [6], and FA, a plant phenolic compound, is significantly known for its antioxidant and free radical-scavenging activities [7]. Although these compounds in nano-formulations show promise for killing drug-resistant bacteria [6], their therapeutic potential is often limited by poor solubility, low bioavailability, and rapid clearance. Additionally, a significant challenge arises with drug-resistant polymicrobial infections, where MRSA and PA synergically form biofilm, which many existing nanoplatform systems, whether synthetic or derived from natural products, have failed to kill.

Recent advances in self-assembled nanotechnology promise to enhance the efficacy of natural products, especially in TCM. Intriguingly, research indicates that some TCM formulas naturally form nanostructures, which are believed to contribute to their enhanced biological function and may reflect the ancient “Jun–Chen–Zuo–[*Shi*]” theory of synergistic composition [6,8]. A key challenge in this area is the effectiveness of drug delivery, for which metal-organic frameworks (MOFs) have shown strong potential [9,10]. MOFs, being three-dimensional porous crystals, are structurally superior to materials such as mesoporous organo-silica (MOS) in preventing drug leakage. Notably, the zirconium-based MOF, UiO-66-NH_2_, stands out for its robust stability, high surface area, and tunable pores, making it an excellent platform for loading and the sustainable release of multiple drugs [11], unlike other MOFs [9,10]. In this framework, the "[*Shi*]" (*courier*) component not only delivers the active agents but also harmonizes their interactions, guides them to the pathological site, and stabilizes the formula. Translating this concept into materials design, we employ UiO-66-NH_2_ not as a passive carrier but as an active "[*Shi*]" that embodies these principles. Its stimuli-responsive release ensures targeted delivery to the infected wound microenvironment; its porous structure and surface functionalization enable co-loading and stabilization of both BBR and FA; and its degradation profile aligns with TCM's emphasis on safety. This approach advances beyond traditional drug delivery by incorporating TCM-inspired synergy directly into the nanomaterial's design.

**Scheme 1:**
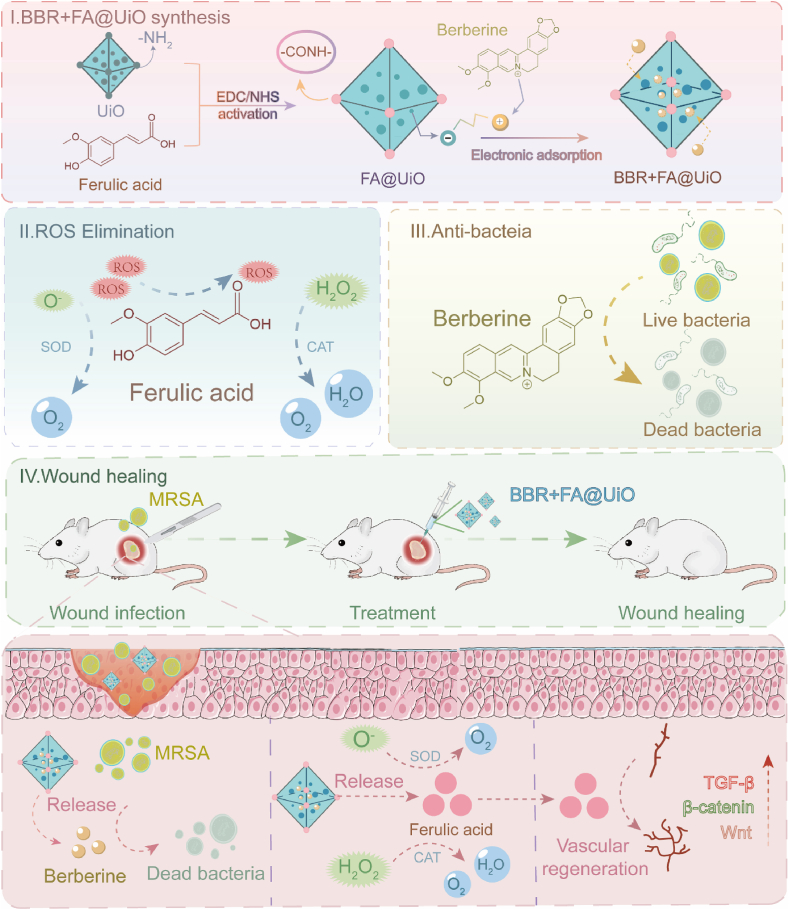
Schematic illustration of the BBR + FA@UiO mechanism for synergistic chronic infected wound therapy.BBR acts as the "Jun" (monarch), FA as the "Chen/Zuo" (minister/assistant), and UiO-66-NH_2_ as the "[*Shi*]" (courier), integrating Traditional Chinese Medicine (TCM) with MOF technology for targeted infection control, oxidative stress resolution, and tissue regeneration.

Building on this evidence, we hypothesize that a novel nanocomposite leveraging the “Jun–Chen–Zuo–[*Shi*]” synergy, with BBR as "Jun," FA as "Chen/Zuo," and the UiO-66-NH_2_ delivery system as "[*Shi*]", can break the destructive cycle of infection and oxidative stress, thereby orchestrating a conducive microenvironment for robust tissue regeneration. Herein, we developed a novel nanotherapeutic, a zirconium-based MOF integrating BBR and FA (BBR + FA@UiO), for the management of multi-resistant chronic wound infections (Scheme 1). This strategy merges TCM principles with modern nanomaterials to create a multifunctional platform that simultaneously targets infection, mitigates oxidative stress, and promotes tissue regeneration in wound care. This innovative combination offers a new perspective on treating difficult-to-heal wounds, which provides insights for designing and improving biomimetic nanomulti-target drugs to maximize therapeutic efficacy.

## 2 Results and discussion

### 2.1 Fabrication and structural characterization of BBR + FA@UiO

**Fig. 1:**
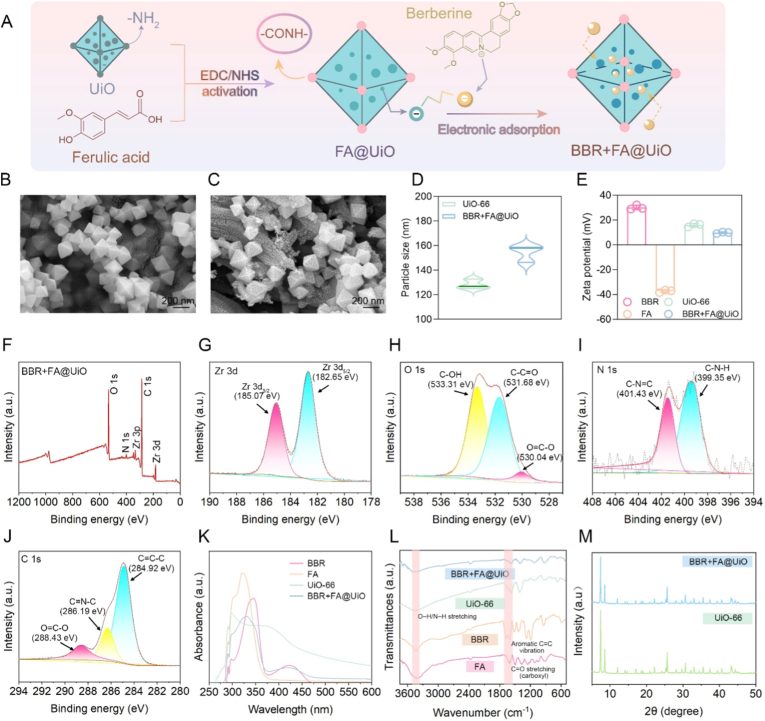
Fabrication and characterization of BBR + FA@UiO nanocomposites. (A) Schematic illustration of the fabrication process and working principle of BBR + FA@UiO. (B, C) SEM images of UiO-66-NH_2_ and BBR + FA@UiO. Scale bar = 200 nm. (D) Dynamic light scattering (DLS) analysis of UiO-66-NH_2_ and BBR + FA@UiO. (E) Zeta potential values of BBR, FA, UiO-66-NH_2_, and BBR + FA@UiO. (F–J) XPS spectra of BBR + FA@UiO: (F) survey spectrum; (G) Zr 3d, (H) O 1s, (I) N 1s, and (J) C 1s core-level spectra. (K) UV–vis absorption spectra of BBR, FA, UiO-66-NH_2_, and BBR + FA@UiO. (L) FT-IR spectra of BBR, FA, UiO-66-NH_2_, and BBR + FA@UiO. (M) XRD patterns of UiO-66-NH_2_ and BBR + FA@UiO.

BBR + FA@UiO nanocomposite was synthesized by covalently bonding ferulic acid (FA) to amino-functionalized UiO-66-NH_2_ via EDC/NHS-mediated amidation, followed by physical adsorption of BBR (Fig. 1A). This combined covalent and non-covalent approach creates a system that harnesses the antioxidant power of FA and the antibacterial effect of BBR within a single, stable metal-organic framework (MOF), promising synergistic action against drug-resistant infection and for tissue repair [12]. SEM analysis revealed that the material retained uniform octahedral crystals after dual-drug loading (Fig. 1B and C), demonstrating the preservation of the MOF's structural integrity. This morphological stability is a known characteristic of UiO-66-NH_2_ MOFs, which are renowned for their high chemical and mechanical robustness [13,14]. Dynamic light scattering showed a slight increase in hydrodynamic size (Fig. 1D), attributed to the surface modification and pore filling. Zeta potential analysis shifted from the positive potential of BBR and the negative potential of FA to near neutral for BBR + FA@UiO (Fig. 1E), indicating successful co-loading and a balanced surface charge that promotes dispersion stability. The shift from positive (UiO-66-NH_2_) to negative (FA@UiO) and finally to near-neutral zeta potential (BBR + FA@UiO) reflects successful FA grafting and subsequent BBR adsorption. The near-neutral surface charge may enhance colloidal stability in physiological environments and favor cellular uptake by minimizing non-specific electrostatic interactions, thereby promoting targeted delivery in wound microenvironments.

XPS confirmed the presence of Zr, O, N, and C and showed the Zr–O coordination environment remained intact after modification (Fig. 1F–J and Fig. S1). High-resolution spectra of O 1s, N 1s, and C 1s displayed signal peaks at ∼531–532 eV (C=O), ∼399 eV (C–N–H), and ∼288 eV (O–C=O), consistent with amide bond formation between FA and the framework while retaining the original amino and aromatic features of UiO-66-NH_2_. These spectral shifts confirm the successful covalent grafting of FA onto the MOF without compromising its chemical integrity [15]. Further evidence comes from UV–vis spectra, which showed the preservation of the absorptions characteristic of both BBR and FA, confirming the incorporation of both drugs (Fig. 1K). FT-IR spectra of BBR + FA@UiO exhibited characteristic absorption bands corresponding to O–H/N–H stretching (∼3300 cm^−1^), C=O stretching of carboxyl groups (∼1680–1700 cm^−1^), and aromatic C=C vibrations (∼1600 cm^−1^), indicating the successful incorporation of FA into the UiO-66-NH_2_ framework.

Furthermore, XRD patterns confirmed that the MOF's crystallinity was preserved post-modification (Fig. 1M). Quantitative analysis revealed efficient drug loading content (LC) and loading efficiency (LE) for BBR and FA in BBR + FA@UiO, with BBR exhibiting an LC of 24.76 % and an LE of 59.1 %. In contrast, the LC (32.4 %) and LE (77.4 %) for FA are consistent with its covalent incorporation into the MOF carrier. These values confirm successful drug loading into the MOF and fall within the typical range reported for small-molecule drugs loaded into UiO-66-NH_2_ MOF systems, highlighting the different interaction modes of FA and BBR with the system's framework. Finally, the nanocomposite exhibited excellent colloidal stability over 7 days in physiologically relevant media, including water, PBS, saline (0.9 % NaCl), and 10 % FBS-supplemented DMEM, as indicated by the hydrodynamic diameter measured by DLS (Fig. S2). Specifically, in aqueous suspension, the hydrodynamic diameter of BBR + FA@UiO remained stable, with less than a 10 % change over 7 days, confirming its structural integrity over a timeframe relevant to wound healing applications. This stability is a crucial requirement for *in vivo* applications. Together, these data demonstrate the successful fabrication of a stable dual-drug nanoplatform in which FA is covalently anchored, and BBR is physically adsorbed onto the UiO-66-NH_2_ MOF. This BBR + FA@UiO nanocomposite retains its crystallinity and chemical structure, making it a suitable candidate for further investigations into controlled drug release and synergistic antibacterial-antioxidant applications [16].

### 2.2 BBR + FA@UiO exhibits potent dual antibacterial and antibiofilm activity

**Fig. 2:**
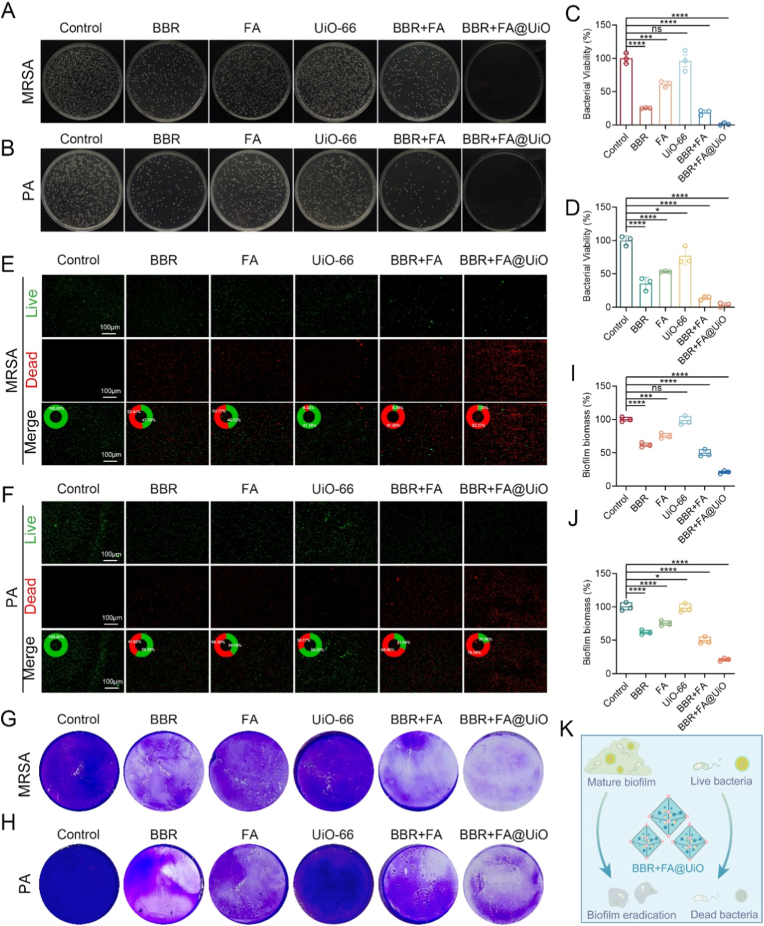
Antibacterial and antibiofilm activity of BBR + FA@UiO. (A, B) Representative colony formation images of MRSA (A) and *PA* (B) after different treatments. (C, D) Quantitative bacterial viability of MRSA (C) and PA (D). (E, F) Live/dead fluorescence staining of MRSA (E) and PA (F). Green fluorescence indicates live bacteria and red fluorescence indicates dead bacteria. (G, H) Crystal violet staining of MRSA (G) and PA (H) biofilms. (I, J) Quantitative analysis of biofilm biomass in MRSA (I) and PA (J). (K) Schematic illustration of the antibacterial mechanism of BBR + FA@UiO. Data are presented as mean ± SD (n = 3); ns, not significant; *∗p < 0.05, ∗∗p < 0.01, ∗∗∗p < 0.001, ∗∗∗∗p < 0.0001.*

The clinical translation of the nanocomposite hinges on its demonstrated efficacy against both bacteria and biofilms. A major hurdle in treating infections is the formation of biofilms, wherein bacteria produce a protective layer of extracellular polymeric substances (EPS) that block antibiotics and strengthen bacterial resistance, leading to persistent and chronic infections [9,17]. Thus, we evaluated the antibacterial and antibiofilm efficacy of the BBR + FA@UiO nanocomposite against two clinically relevant pathogens, MRSA and PA. Colony formation assays demonstrated that treatment with BBR + FA@UiO completely eradicated both MRSA (Fig. 2A) and PA (Fig. 2B), an effect that was substantially superior to that of any individual component (BBR, FA, UiO-66) or the physical mixture (BBR + FA). Quantitative analysis confirmed a statistically significant reduction in bacterial viability (>98 %), with the BBR + FA@UiO group exhibiting the most significant suppression for both strains (Fig. 2C and D).

Live/dead fluorescence staining (Fig. 2E and F) further confirmed that the BBR + FA@UiO group completely eradicated bacteria (intense red fluorescence), whereas the control groups showed live bacteria (green fluorescence). The Live/Dead assay, which utilizes propidium iodide (PI) uptake, directly indicates loss of membrane integrity in non-viable bacteria. The significant increase in red fluorescence (PI-positive cells) in the BBR + FA@UiO treatment group strongly supports membrane disruption as a key mechanism of action. Quantitative analysis revealed that more than 90 % of the MRSA (Figs. 2E) and 80 % of the PA bacteria (Fig. 2F) became non-viable following treatment with PCNZnCy. Given the critical role of biofilms in chronic infections, we next assessed the nanocomposite's anti-biofilm capacity. Crystal violet staining revealed that BBR + FA@UiO drastically inhibited biofilm formation in both MRSA (Fig. 2G) and PA (Fig. 2H), resulting in visibly less stained biomass. Similarly, the crystal violet assay demonstrates inhibition of biofilm formation, consistent with FA interfering with biofilm-related enzymes and quorum sensing. Quantitative analysis of biofilm biomass confirmed that treatment with BBR + FA@UiO completely disrupted the dense biofilm and substantially reduced biomass compared with all other control groups (Fig. 2I and J). These findings suggest that BBR + FA@UiO operates through a synergistic mechanism (Fig. 2K), in which the UiO-66-NH_2_ metal-organic framework (MOF) serves as a co-delivery vehicle for BBR, thereby facilitating rapid bactericidal action. Concurrently, FA contributes both antibacterial and antioxidant properties. The limited antibacterial and antioxidant effects of free BBR or FA alone are likely due to poor solubility, rapid clearance, and absence of targeted delivery. In contrast, co-loading within UiO-66-NH_2_ enhances compound stability, prolongs wound-site retention, and enables pH/ROS-responsive co-release. This ensures that BBR and FA act synergistically in time and space, with BBR providing an immediate bactericidal effect and FA sustaining antioxidant support, thereby optimizing therapeutic efficacy.

Mechanistically, BBR, a natural isoquinoline alkaloid, exerts antibacterial effects primarily by disrupting bacterial cell membrane integrity, inhibiting DNA replication, and interfering with protein synthesis through interactions with bacterial topoisomerases and gyrases [6,18]. FA, a phenolic acid, contributes to antibacterial activity via membrane perturbation, inhibition of key enzymatic functions (e.g., ATP synthase and biofilm-related enzymes), and disruption of quorum sensing [19]. The combination of BBR and FA within the UiO-66 framework not only enhances their individual antibacterial properties but also creates a multi-target strategy that reduces the likelihood of resistance development and addresses both planktonic and biofilm-associated bacteria. *Given that chronic wounds often harbor polymicrobial communities, future research should evaluate BBR + FA@UiO against mixed-species biofilms, such as MRSA + PA, to better* mimic *clinical infection.* Additionally, future studies employing TEM/SEM imaging or quantitative membrane permeability assays, such as DiBAC4(3) or PI uptake kinetics, could further provide deeper insights into ultrastructural changes and dynamic membrane damage, offering a more detailed mechanistic understanding of the synergistic action. Collectively, BBR + FA@UiO is a promising nano-antibacterial platform that merges the benefits of natural compounds with advanced nanotechnology, offering a versatile mechanism of action with potential applications against multidrug-resistant pathogens.

### 2.3 Drug release and antioxidant performance of BBR + FA@UiO

**Fig. 3:**
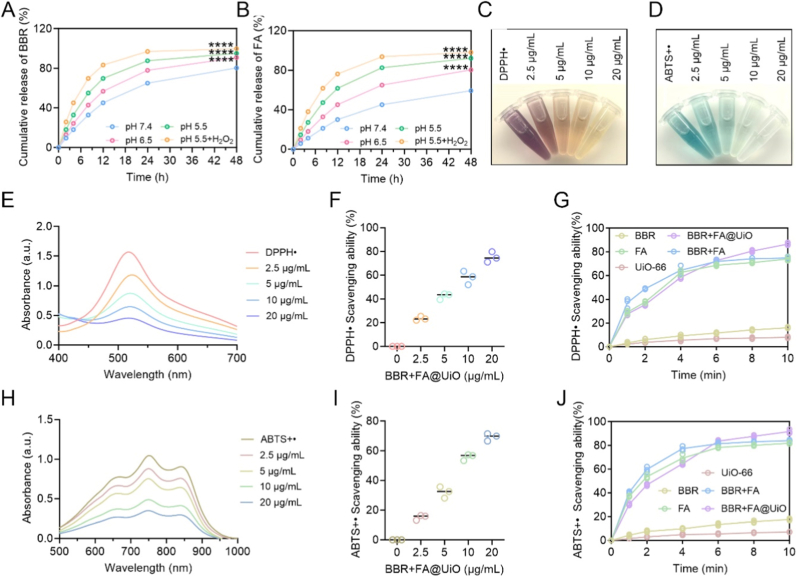
Drug release and antioxidant activity of BBR + FA@UiO. (A, B) Cumulative release profiles of BBR and FA from BBR + FA@UiO. (C, D) DPPH• and ABTS•+ solutions after treatment with different concentrations of BBR + FA@UiO. (E, H) UV–vis spectra of DPPH• and ABTS•+ radicals with various concentrations of BBR + FA@UiO. (F, I) Free-radical scavenging efficiency of BBR + FA@UiO at different concentrations. (G, J) DPPH• and ABTS•+ scavenging kinetics of UiO-66-NH_2_, BBR, FA, BBR + FA, and BBR + FA@UiO. Data are presented as mean ± SD (n = 3); ns, not significant; *∗p < 0.05, ∗∗p < 0.01, ∗∗∗p < 0.001, ∗∗∗∗p < 0.0001*.

Evaluation of drug release and antioxidant performance of the nanomaterials is crucial for clinical translation [19]. A defining feature of advanced wound-healing systems is their ability to respond to pathological stimuli, such as pH and reactive oxygen species (ROS) gradients, enabling coordinated antibacterial and anti-inflammatory effects [20,21]. The BBR + FA@UiO dual-drug system exemplifies this principle, demonstrating both pH- and ROS-responsive release and potent antioxidant activity (Fig. 3A and B). While BBR and FA release slowly at physiological pH (7.4), they increase significantly under the mildly acidic and ROS-rich conditions typical of a wound site (pH 6.5–5.5 with H_2_O_2_). In a simulated severe infection microenvironment (pH 5.5 + H_2_O_2_), cumulative release reaches ∼90–100 % within 48 h. This rapid release is consistent with a mechanism involving protonation of amino groups under acidic conditions and oxidative cleavage of chemical bonds (e.g., amide or coordination bonds) in the presence of H_2_O_2_, thereby destabilizing the framework and enhancing drug diffusion. Similar pH- and ROS-responsive decomposition mechanisms have been spectroscopically demonstrated in amino-functionalized UiO-66 analogues, where acid-induced protonation and ROS-mediated bond cleavage jointly promote payload release [22]. Several additional studies indicated that in acidic environments, MOFs break down and collapse, releasing their trapped contents at specific locations due to protonation of carboxylate groups and surface functionalities [23,24]. While direct spectroscopic confirmation of these chemical changes in BBR + FA@UiO would further validate the mechanism, the observed release kinetics strongly support a synergistic, stimulus-triggered release process that aligns with the pathological wound microenvironment. This release kinetics suggests a therapeutic strategy where BBR rapidly drives antibacterial action during early infection, while FA is released gradually to alleviate oxidative stress and support tissue regeneration [12].

The antioxidant performance of BBR + FA@UiO was assessed using DPPH• and ABTS•+ radical scavenging assays. The result revealed that BBR + FA@UiO visibly faded both radicals (Fig. 3C and D), and their absorbance values declined as the nanocomposite concentration increased (Fig. 3E–H), confirming effective radical scavenging. Significant dose-dependent radical scavenging was further observed (Fig. 3F–I), and kinetic data showed rapid action, achieving over 80 % scavenging within 10 min (Fig. 3G–J), exceeding that of BBR, FA, and their physical mixture (BBR + FA). The antioxidant performance of BBR + FA@UiO was further extended to physiologically relevant ROS. The nanocomposite exhibited efficient, dose-dependent scavenging of hydrogen peroxide (H_2_O_2_) and hydroxyl radicals (•OH) (Fig. S3). This broad-spectrum ROS-scavenging capability is particularly relevant to the wound microenvironment, where H_2_O_2_ acts as a signaling molecule and •OH causes severe oxidative damage to lipids, proteins, and DNA. The observed performance likely stems from the combined redox properties of FA, the synergistic effect of BBR, and the high surface exposure of active groups within the UiO-66-NH_2_ framework [25]. Overall, BBR + FA@UiO integrates intelligent, pH/ROS-triggered release with potent antioxidant activity, creating a dynamic therapeutic platform for stage-specific management of infected chronic wounds. While the current data robustly demonstrate the critical, microenvironment-responsive release necessary for therapeutic application, a more detailed mechanistic analysis dissecting the individual and synergistic contributions of pH and ROS to drug liberation represents a valuable direction for subsequent investigation. Future studies using *in situ* spectroscopic techniques such as FT-IR or XPS to monitor chemical changes in the MOF framework upon exposure to acidic/ROS conditions would provide direct verification of the proposed release mechanism.

### 2.4 BBR + FA@UiO mitigates oxidative stress and preserves mitochondrial function via Nrf2/Keap1 pathway activation

**Fig. 4:**
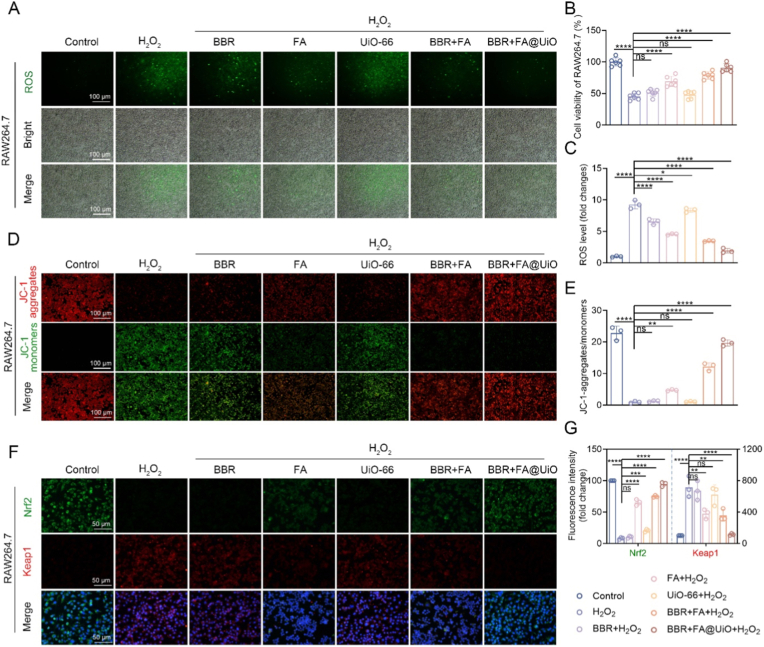
BBR + FA@UiO alleviates oxidative stress and mitochondrial dysfunction through Nrf2/Keap1 signaling. (A) DCFH-DA assays showing fluorescence images of intracellular ROS levels in RAW 264.7 cells under various treatments. (B) Cell Counting Kit-8 (CCK-8) assay for Cell viability of RAW264.7 cells. (C) Quantification of ROS levels. (D) JC-1 staining images showing mitochondrial membrane potential. Red fluorescence for JC-1 aggregates (healthy mitochondria) and green fluorescence for JC-1 monomers (depolarized mitochondria). (E) Quantification of JC-1 aggregate/monomer ratio. (F) Immunofluorescence staining of Nrf2 (green) and Keap1 (red) in RAW264.7 cells, nuclei counterstained with DAPI (blue). (G) Quantification of Nrf2 and Keap1 fluorescence intensity. Data are presented as mean ± SD (n = 3). Statistical analysis: ns, not significant; *∗p < 0.05, ∗∗p < 0.01, ∗∗∗p < 0.001, ∗∗∗∗p < 0.0001*.

To investigate whether BBR + FA@UiO protects cells from oxidative stress, H_2_O_2_-stimulated RAW264.7 cells were employed as a model. As shown in Fig. 4A–C, H_2_O_2_ markedly increased ROS accumulation and reduced cell viability, confirming oxidative injury. Treatment with free BBR, FA, or UiO-66-NH_2_ provided only limited protection, while the BBR + FA mixture exerted moderate antioxidant activity. In contrast, BBR + FA@UiO significantly reduced ROS levels and restored cell viability to near-control levels, demonstrating a strong synergistic protective effect. This finding is significant, as excessive ROS production from both infiltrating immune cells and bacterial metabolism in infectious microenvironments can cause substantial host tissue damage, impair healing, and perpetuate a cycle of inflammation [26]. The ability of BBR + FA@UiO to effectively scavenge ROS suggests its potential to protect host cells and create a more favorable environment for tissue repair, a feature often lacking in conventional antimicrobials or TCM formulations [18,27].

Given the central role of mitochondria in redox homeostasis [26], we assessed mitochondrial function using JC-1 staining. H_2_O_2_-exposed cells showed a greater proportion of green fluorescence (JC-1 monomers), which gradually decreased in the other groups, suggesting mitochondrial depolarization (Fig. 4D). In contrast, cells treated with BBR + FA@UiO exhibited a strong red fluorescence signal, indicative of JC-1 aggregates and a healthy, high mitochondrial membrane potential (ΔΨm). Quantification of the JC-1 aggregate/monomer ratio confirmed that BBR + FA@UiO treatment most effectively preserved mitochondrial membrane integrity (Fig. 4E). This demonstrates that BBR + FA@UiO maintains cellular viability and function, which are critical for effective immune responses and tissue regeneration at the site of infection.

To elucidate the molecular mechanism underlying this antioxidant activity, we investigated the Nrf2/Keap1 signaling pathway. Immunofluorescence staining revealed that BBR + FA@UiO treatment substantially enhanced the nuclear translocation of Nrf2 while promoting the cytoplasmic retention of its negative regulator, Keap1 (Fig. 4F). Quantitative analysis of fluorescence intensity confirmed a significant increase in nuclear Nrf2 and a corresponding decrease in Keap1 signal in the BBR + FA@UiO group (Fig. 4G). This finding implies that BBR exerts its effects by modulating the Nrf2/Keap1 pathway, a primary cellular defense system against oxidative stress [28]. Mechanistically, BBR binds to Keap1, thereby stabilizing Nrf2 and facilitating its nuclear translocation [29]. Inside the nucleus, Nrf2 initiates transcription of genes encoding antioxidant and detoxifying enzymes, including HO-1 and NQO-1, thereby enhancing cellular protection [30].

Moreover, FA operates through a complementary mechanism [19], synergistically enhancing this process to upregulate genes that mitigate oxidative damage and restore mitochondrial function [31,32]. The co-delivery of BBR and FA within the UiO-66 MOF is pivotal, enabling their concerted action on this critical signaling axis. UiO-66-NH_2_ serves as an exemplary “[*Shi*]” (courier) in the theory of TCM “Jun–Chen–Zuo–[*Shi*]” framework due to its robust stability, high porosity for multi-drug loading, surface functionalizability, and stimuli-responsive release. These properties allow it to guide and harmonize the actions of BBR (‘Jun’) and FA (‘Chen/Zuo’), protecting them from degradation, directing them to the infected wound microenvironment, and coordinating their release to achieve integrated antibacterial, antioxidant, and pro-regenerative effects. This dual-targeting strategy simultaneously confronts two key pathological elements of complex infections, representing a holistic and potent therapeutic approach. Consequently, this research highlights a shift in antimicrobial development toward multifunctional nanomaterials designed to both eradicate pathogens and support the recovery of host tissues.

### 2.5 BBR + FA@UiO alleviates oxidative stress and promotes proliferation and migration of HUVECs under H_2_O_2_-induced injury

**Fig. 5:**
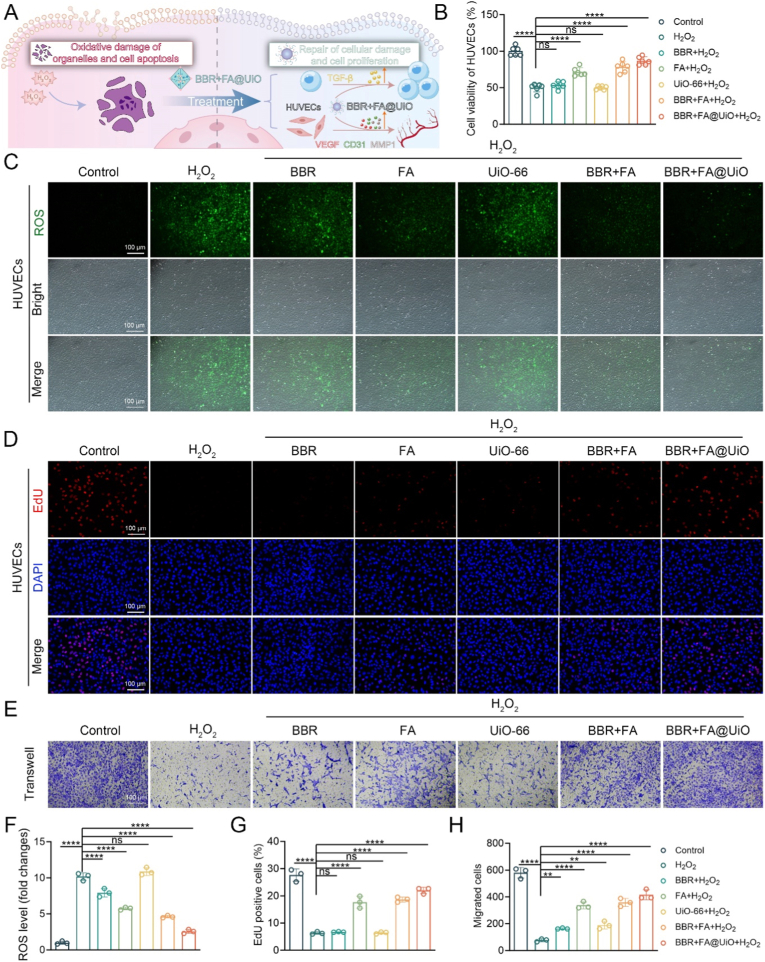
BBR + FA@UiO alleviates oxidative stress and promotes proliferation and migration of HUVECs under H_2_O_2_-induced injury. (A) Schematic illustration showing the protective and reparative effects of BBR + FA@UiO nanocomposite on H_2_O_2_-induced oxidative injury in HUVECs. (B) Cell viability of HUVECs after different treatments assessed by CCK-8 assay (n = 6). (C) DCFH-DA assays showing the fluorescence images of intracellular ROS levels. (D) EdU staining showing proliferating HUVECs (red) with DAPI nuclear counterstain (blue). (E) Transwell assay images illustrating HUVEC migration after different treatments. (F–H) Quantification of ROS levels (F), EdU-positive cell ratio (G), and migrated cell numbers (H). Data are presented as mean ± SD (n = 3–5). Statistical significance: ns, not significant; *∗p < 0.05, ∗∗p < 0.01, ∗∗∗p < 0.001, ∗∗∗∗p < 0.0001.*

Building on the above results, we next investigated the proliferation and migration of HUVECs under H_2_O_2_-induced injury **(**Fig. 5A**).** The H_2_O_2_ stimulation model is widely used to replicate oxidative endothelial damage, characterized by overproduction of ROS, impairment of mitochondrial function, and a decline in angiogenic potential [19]. This feature aligns with oxidative stress conditions in infected wounds, where elevated H_2_O_2_ levels compromise endothelial integrity and inhibit key pro-angiogenic pathways, including VEGF and eNOS. Consistent with this evidence, H_2_O_2_ stimulation significantly reduced cell viability and proliferation, increased intracellular ROS, and hindered cell migration (Fig. 5B–H). Treatment with free BBR, FA, or the UiO-66 carrier alone partially mitigated these adverse outcomes, but recovery was limited. This transient action and poor retention of the free drugs likely constrained their efficacy, underscoring the benefit of a sustained-release delivery system. Conversely, BBR + FA@UiO elicited a robust therapeutic response, effectively restoring viability to control levels and substantially lowering ROS (Fig. 5C–F). This suggests that UiO-66-NH_2_ MOF enables the controlled co-delivery of antioxidants, thereby maintaining redox homeostasis and promoting endothelial repair, aligning with previous applications of MOFs in vascular regeneration [16]^.^

Further EdU and transwell assays confirmed that BBR + FA@UiO treatment led to a significantly greater recovery of both proliferative and migratory capacities than any other group (Fig. 5D–H). These findings suggest that the UiO-66 MOF promotes a sustained synergistic action between BBR and FA. By concurrently scavenging ROS and reactivating critical repair mechanisms, the BBR + FA@UiO nanocomposite strategy shows considerable promise for promoting angiogenesis and tissue regeneration in chronic wound healing [33].

### 2.6 BBR + FA@UiO accelerates wound healing and suppresses bacterial infection *in vivo*

**Fig. 6:**
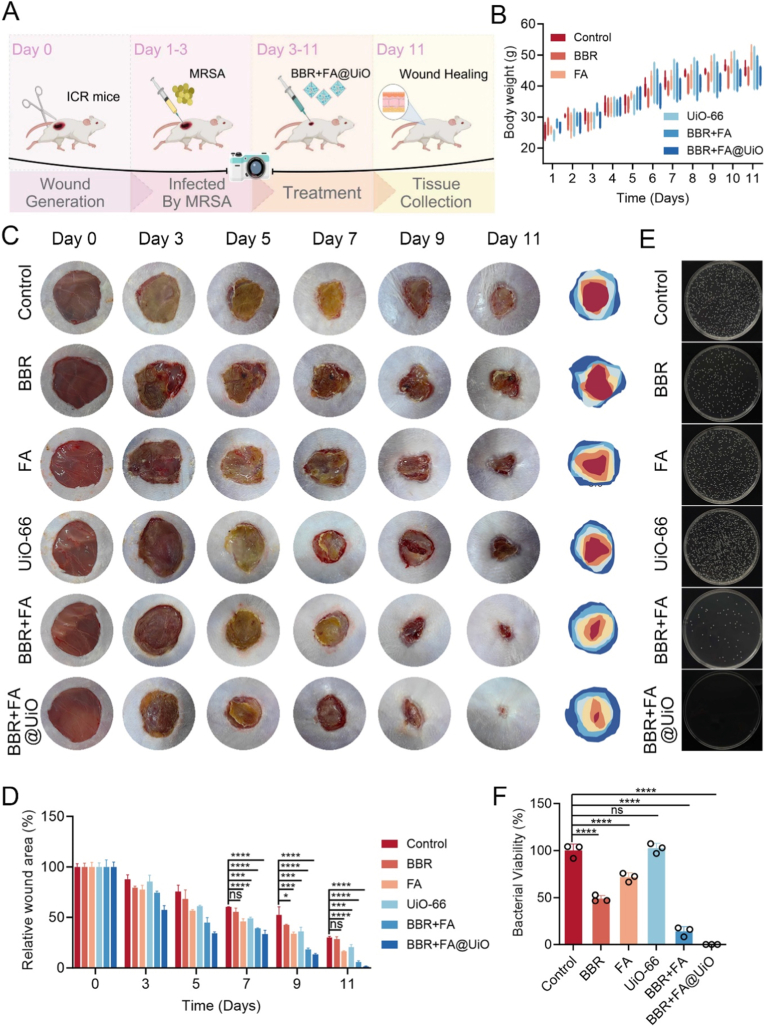
*In vivo* evaluation of BBR + FA@UiO in a MRSA-infected wound model. (A) Schematic illustration of the experimental timeline. (B) Body weight changes of mice during 11 days of treatment. (C) Images of wound healing progression at days 0, 3, 5, 7, 9, and 11 in different treatment groups. (D) Quantitative analysis of relative wound area. (E) Bacterial colony images from wound homogenates. (F) Quantification of bacterial viability from wound tissues. Data are presented as mean ± SD (n = 5); Statistical significance: ns, not significant; *∗p < 0.05, ∗∗p < 0.01, ∗∗∗p < 0.001, ∗∗∗∗p < 0.0001*.

The *in vivo* effectiveness of BBR + FA@UiO was investigated using a standard MRSA-infected wound model in mice (Fig. 6A). MRSA was chosen for its high prevalence in chronic wounds, including biofilm formation, inflammation, and impaired healing, and for its well-established murine model that allows reproducible assessment of antibacterial and healing effects. The *in vivo* dose was chosen based on the strong *in vitro* antibacterial effectiveness of BBR + FA@UiO. To ensure a strong therapeutic effect in the complex wound environment, considering factors like wound exudate dilution, biofilm penetration, and sustained activity, we topically administered a dose of 1 mg/mL (100 μg per 100 μL). This is a 2- to 4-fold higher concentration than the fully effective *in vitro* level, which is a standard and cautious approach for translating topical antimicrobial effects from *in vitro* to *in vivo* models [19,34]. This dose was administered every 2 days to maintain adequate drug levels and to follow established protocols for topical wound treatments in mouse models [34]. All treatment groups showed similar weight gain over the investigation, indicating good biocompatibility and minimal systemic toxicity (Fig. 6B). Gross wound inspection revealed distinct patterns of healing. Controls displayed persistent exudation and inflammation with poor healing, while those treated with free BBR showed a modest reduction in infection, consistent with its documented anti-biofilm activity [35]. FA alone offered minimal antibacterial benefit but is recognized for mitigating oxidative damage and supporting angiogenesis [36], while the UiO-66-NH_2_ MOF had no significant effect. The combined free drugs (BBR + FA) improved wound contraction relative to individual components, but the most pronounced and rapid healing was seen with BBR + FA@UiO, showing near-complete closure by day 11 (Fig. 6C and D).

Bacterial counts from wound homogenates corroborated the above observations. Dense colonies were recovered from control and individual component groups; BBR + FA reduced the bacterial load, and BBR + FA@UiO nearly eradicated MRSA, with only a few colonies remaining (Fig. 6E). Quantification confirmed that BBR + FA@UiO reduced viable bacteria by over 90 % compared to the control group (Fig. 6F), a log-scale reduction consistent with other advanced local antimicrobial strategies [34].

The superior efficacy of BBR + FA@UiO is likely due to a multi-faceted mechanism, including the direct antibacterial action of BBR that synergizes with FA's antioxidant and pro-regenerative effects, and the UiO-66-NH_2_ MOFs provide controlled, sustained release of the drugs in response to the wound's specific pH/ROS microenvironment. These findings are consistent with previous studies on pH/ROS-responsive MOF systems for infection control and the demonstrated biocompatibility and controllable drug release of UiO-66-NH_2_ MOFs [37,38]. Together, BBR + FA@UiO represents a promising therapeutic platform that combines the efficacy of traditional drugs with advanced, responsive delivery to treat complex infected wounds effectively.

### 2.7 BBR + FA@UiO promotes angiogenesis and collagen deposition to accelerate wound tissue regeneration

**Fig. 7:**
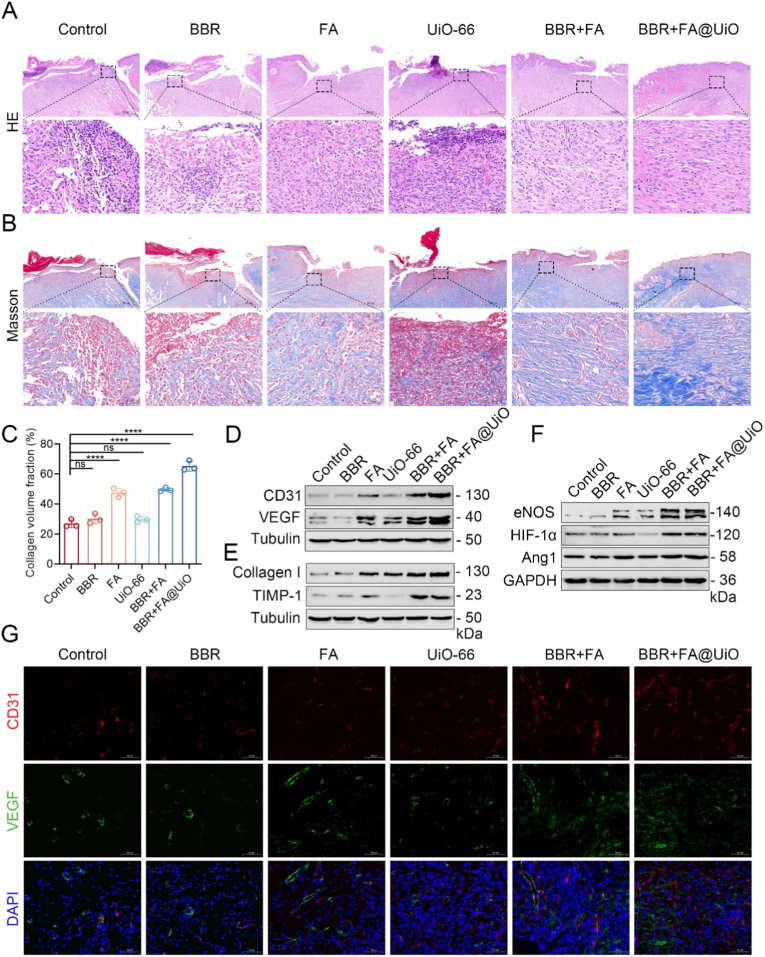
BBR + FA@UiO enhances tissue regeneration in infected wounds. (A) H&E-stained wound tissue sections. (B) Masson's trichrome staining of the collagen deposition in wound tissues. (C) Quantitative analysis of collagen volume fraction. (D) Western blot analysis of angiogenesis-related proteins (CD31, VEGF). (E) Western blot analysis of extracellular matrix (ECM)-related markers (Collagen I, TIMP-1). (F) Western blot of vascular regeneration and oxygen homeostasis markers (eNOS, HIF-1α, Ang1). (G) Immunofluorescence staining of CD31 (red) and VEGF (green) in wound sections. Data are presented as mean ± SD (n = 3); Statistical significance: ns, not significant; *∗p < 0.05, ∗∗p < 0.01, ∗∗∗p < 0.001, ∗∗∗∗p < 0.0001*.

Recent studies have highlighted the potential of MOF nanocomposites to simultaneously reduce inflammation and promote collagen deposition and angiogenesis during wound healing [39]. To further investigate the regenerative effects of BBR + FA@UiO, we performed histological and molecular analyses on wound tissues. H&E staining showed persistent epithelial defects and heavy inflammatory infiltration in the control, FA, and UiO-66 groups. BBR + FA treatment produced partial re-epithelialization, while BBR + FA@UiO-treated wounds exhibited almost complete epithelial coverage with markedly less inflammatory cell presence (Fig. 7A). This anti-inflammatory effect was confirmed at the molecular level, with enzyme-linked immunosorbent assay (ELISA) showing a significant reduction in key pro-inflammatory cytokines (TNF-α, IL-1β, IL-6) in wound homogenates from the BBR + FA@UiO group (Fig. S4). This finding suggests that BBR + FA@UiO may attenuate the early inflammatory phase, potentially by inhibiting key pro-inflammatory mediators such as IL-1β and TNF-α and suppressing the NF-κB pathway, a mechanism observed in other antioxidant-functionalized MOF systems [40]. Masson's trichrome staining revealed scarce collagen deposition in control and individual component groups, whereas BBR + FA@UiO markedly increased collagen fiber content and alignment, indicating advanced matrix remodeling (Fig. 7B and C). Collagen I, as the predominant structural protein in the dermal ECM, provides tensile strength and serves as a scaffold for cell migration and tissue regeneration [41]. TIMP-1, a tissue inhibitor of metalloproteinases-1, helps maintain ECM homeostasis by preventing excessive degradation by matrix metalloproteinases (MMPs), thereby supporting stable collagen accumulation and maturation [42]. Additionally, collagen fibers in the BBR + FA@UiO group exhibited enhanced crosslinking density and similar orientation, hallmarks of a mature, mechanically stable ECM [43].

Moreover, Western blot analysis confirmed a significant increase in the expression of the angiogenesis-related proteins CD31 and VEGF in the BBR + FA@UiO group relative to other treatments (Fig. 7D and Fig. S5). Such an enhancement is a recognized effect of nano-platforms that shift the wound microenvironment toward a pro-angiogenic state [44]. The coordinated increases in CD31, VEGF, eNOS, and HIF-1α imply activation of the well-established HIF-1α/VEGF/eNOS signaling pathway, a critical driver of endothelial cell proliferation and new blood vessel maturation [45]. Furthermore, increased levels of the extracellular matrix proteins Collagen I and TIMP-1 confirmed enhanced structural repair (Fig. 7E and Fig. S6). By concurrently promoting Collagen I synthesis and inhibiting its degradation via TIMP-1, BBR + FA@UiO shifts the wound environment from a catabolic to a robust anabolic state, thereby facilitating the accumulation of a strong, organized collagen matrix. The increased expression of eNOS, HIF-1α, and Ang1 following BBR + FA@UiO treatment further corroborated the strengthened pro-angiogenic signaling (Fig. 7F and Fig. S7). This is consistent, as no documented role of MOF-based materials has been reported in modulating redox balance to stabilize HIF-1α, which in turn upregulates downstream effectors such as VEGF and Ang1 to orchestrate vascular growth. Supporting these findings, immunofluorescence staining significantly illustrated strong positive signals for both CD31 and VEGF in the BBR + FA@UiO-treated wounds, indicating a high degree of new blood vessel formation compared with other groups (Fig. 7G and Fig. S8).

The data collectively demonstrate that BBR + FA@UiO effectively combats infection and actively promotes a regenerative wound environment by stimulating angiogenesis and collagen deposition. These synergistic outcomes are likely due to the combined antibacterial and antioxidant properties of BBR and FA, with the UiO-66-NH_2_ MOFs serving as delivery vehicles that create a controlled microenvironment conducive to endothelial function and collagen assembly.

### 2.8 BBR + FA@UiO promotes wound healing by activating Nrf2/HO-1 antioxidant signaling and tissue repair pathways

**Fig. 8:**
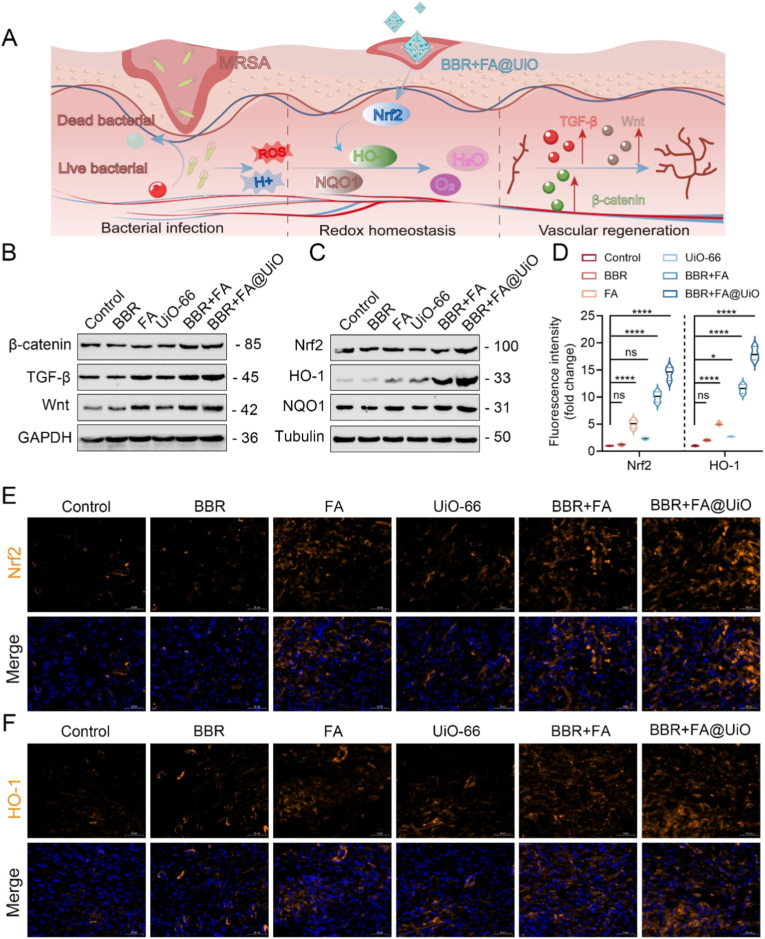
Mechanistic insights into wound healing promoted by BBR + FA@UiO. (A) Schematic illustration of the mechanism insights into wound healing promoted by BBR + FA@UiO**.** (B) Western blot analysis of wound-healing–related proteins (β-catenin, TGF-β, Wnt). (C) Western blot analysis of antioxidant signaling proteins (Nrf2, HO-1, NQO1). (D) Quantitative analysis of fluorescence intensity for Nrf2 and HO-1 immunostaining (corresponding to panels E and F). (E, F) Immunofluorescence staining of Nrf2 (E) and HO-1 (F) in wound tissues. Data are presented as mean ± SD (n = 3); Statistical significance: ns, not significant; *∗p < 0.05, ∗∗p < 0.01, ∗∗∗p < 0.001, ∗∗∗∗p < 0.0001*.

To investigate the molecular mechanisms underlying the enhanced healing seen with BBR + FA@UiO, we examined key repair and antioxidant pathways (Fig. 8A). Western blotting revealed that treatment with BBR + FA@UiO substantially increased levels of β-catenin, TGF-β, and Wnt compared with other groups, consistent with enhanced epithelial regeneration and ECM remodeling (Fig. 8B and Fig. S9). The Wnt/β-catenin pathway is a well-established driver of hair follicle morphogenesis and re-epithelialization [46,47], while TGF-β is a central mediator of fibroblast activation and collagen deposition [48,49]. The activation of the canonical Wnt/β-catenin pathway contributes to wound healing through multiple mechanisms. It promotes epithelial cell proliferation and migration (re-epithelialization), stimulates fibroblast activation and myofibroblast differentiation, and enhances ECM synthesis and remodeling, including the upregulation of Collagen I [47]. Thus, Wnt/β-catenin signaling acts as a central coordinator linking epithelial repair, stromal cell activity, and matrix deposition**.** This finding suggests potential crosstalk between Nrf2 activation and the canonical Wnt/β-catenin and TGF-β/Smad signaling cascades, as ROS suppression by Nrf2 can remove inhibitory constraints on these pro-regenerative pathways.

Given that oxidative stress impedes chronic wound repair [19], we further assessed antioxidant signaling. The result revealed that BBR + FA@UiO markedly increased the expression of Nrf2 and its downstream targets, HO-1 and NQO1, relative to controls and other treatment groups (Fig. 8C and Fig. S10). This result was confirmed by immunofluorescence, revealing faint signals for Nrf2 and HO-1 in the control, FA, and UiO-66 groups, a moderate increase with BBR + FA, and the most intense nuclear and cytoplasmic staining in the BBR + FA@UiO-treated groups (Fig. 8D–F), aligning with previous research, showing that BBR can activate the Nrf2/HO-1 pathway under oxidative stress conditions [30]. Overall, these findings suggest that BBR + FA@UiO promotes bacterial clearance and tissue regeneration by potently activating the Nrf2/HO-1 antioxidant pathway, thereby mitigating oxidative stress. This MOF-based platform, inspired by traditional medicine, offers a unified therapeutic strategy by integrating antibacterial, antioxidant, and tissue-repairing properties.

### 2.9 Biosafety evaluation of BBR + FA@UiO *in vitro* and *in vivo*

**Fig. 9:**
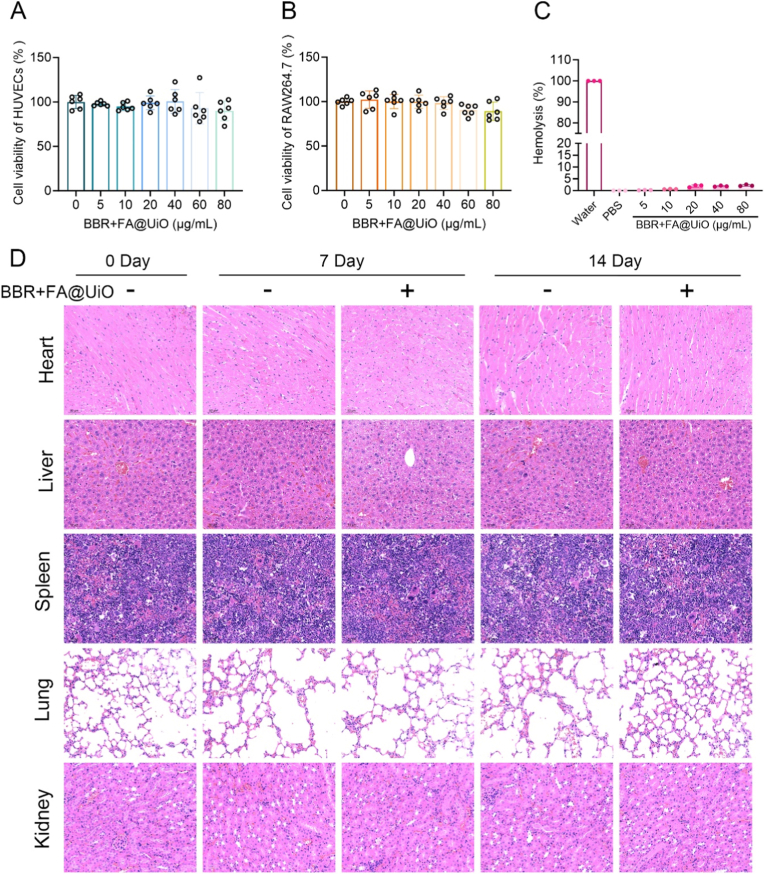
Biosafety evaluation of BBR + FA@UiO *in vitro* and *in vivo*. (A, B) CCK-8 assay showing cell viability of HUVECs (A) and RAW264.7 cells (B) after BBR + FA@UiO treatment (0–80 μg/mL) for 24 h. (C) Hemolysis assay of BBR + FA@UiO at various concentrations. (D) H&E staining images of major organs (heart, liver, spleen, lung, kidney) collected at day 0, day 7, and day 14 after intravenous administration of BBR + FA@UiO. Scale bar = 100 μm.

To explore clinical translational potential, we conducted comprehensive biosafety testing of BBR + FA@UiO *in vitro* and *in vivo* (Fig. 9). CCK-8 assays showed that HUVECs and RAW264.7 cells exhibited high viability (>90 %) at concentrations up to 80 μg/mL, indicating the nanocomposite's low cytotoxicity (Fig. 9A and B). This high cellular tolerance is consistent with the known cytocompatibility of UiO-66-NH_2_ MOFs, which stems from their intrinsic chemical stability and the non-toxic nature of their degradation byproducts [50]. Furthermore, hemolysis testing revealed low red blood cell lysis across all concentrations (<), a level comparable to PBS, demonstrating excellent hemocompatibility (Fig. 9C). Such negligible hemolytic activity is supported by research showing that functionalizing MOF surfaces with biocompatible ligands, such as polyphenols or amino acids, can further improve hemocompatibility [51,52].

Moreover, histological examination of major organs (heart, liver, spleen, lung, kidney) at days 7 and 14 after treatment showed no apparent tissue damage, inflammatory infiltration, or structural abnormalities relative to controls (Fig. 9D). This lack of systemic toxicity suggests that the MOF carrier is safely biodegraded and cleared by the kidneys without inducing an inflammatory response, a documented benefit of nanoscale Zr-MOFs in biomedical contexts [53]. Altogether, these results indicate that BBR + FA@UiO has a favorable safety profile, with low cytotoxicity, negligible hemolysis, and no detectable histopathological abnormalities in major organs. In contrast, the histological assessment suggests an absence of acute systemic toxicity, a comprehensive evaluation, including blood biochemical analysis of liver and kidney function markers, would be necessary in the future to substantiate the systemic safety profile. These findings support the suitability of BBR + FA@UiO for further *in vivo* therapeutic development in infected-wound applications. Subsequent investigations, as suggested for clinical translation of MOF-based agents, should focus on quantifying their pharmacokinetics, biodistribution, and long-term metabolic fate [54].

While the present study significantly confirms the short-term (7 days) and long-term (14 days) biocompatibility and negligible systemic toxicity of BBR + FA@UiO, as with standard nanomaterials, the potential long-term biosafety at 28 days required for MOF-based nanomaterials warrants further investigation using multiple and comprehensive strategies for clinical translation. Key considerations include the *in vivo* degradation kinetics of the UiO-66-NH_2_ MOF, potential accumulation of metal ions or organic linkers in organs, and the immune response to repeated or prolonged exposure. Recent reviews highlight that although Zr-MOFs like UiO-66-NH_2_ exhibit good chemical stability and low acute toxicity, their long-term metabolic fate, renal clearance efficiency, and potential immunogenic effects require systematic evaluation in advanced preclinical models [54]. Future studies should focus on chronic toxicity assessments, biodistribution profiling, and the impact of MOF degradation products on cellular and genomic integrity to ensure safe and sustainable therapeutic applications.

## 3 Conclusion

In summary, we developed a multifunctional nanocomposite, BBR + FA@UiO, designed to fight infection and promote tissue regeneration in chronic, non-healing wounds infected with drug-resistant bacteria. Inspired by the theory of TCM "Jun–Chen–Zuo–[*Shi*]", the system co-delivers an antibacterial "Jun" agent (BBR) and an antioxidant/regenerative "Chen/Zuo" agent (FA). BBR + FA@UiO shows potent dual antibacterial and antibiofilm activity against MRSA and PA. Its stimuli-responsive release mechanism enables precise control over the delivery of therapeutic agents. Functionally, we demonstrated that BBR + FA@UiO activates the Nrf2/Keap1 antioxidant pathway, which preserves mitochondrial function integrity and reduces oxidative stress in immune cells. BBR + FA@UiO also directly supports vascular repair by enhancing endothelial cell proliferation and migration. Crucially, in a murine model of MRSA-infected wounds, BBR + FA@UiO significantly accelerated wound closure through a combination of bacterial eradication, enhanced angiogenesis, and collagen deposition. The activation of key pro-healing pathways (Wnt/β-catenin, TGF-β) and the antioxidant Nrf2/HO-1 axis in healed tissues confirms its role as an active signaling modulator rather than merely a passive drug carrier. Additionally, BBR + FA@UiO nanocomposite exhibited an excellent biosafety profile, with minimal cytotoxicity, hemolysis, and no histopathological signs of organ toxicity. Further blood biochemical analysis in future studies should comprehensively confirm its systemic safety.

These data underscore the importance of designing delivery systems where the carrier itself is entirely transformed into a therapeutic agent, thereby maximizing treatment effectiveness. While the present work focused on chronic wound infection models, the underlying theory of TCM offers a promising framework for developing drug delivery systems for broader diseases. *Future studies should further investigate these findings in polymicrobial and multidrug-resistant preclinical infection models to validate their therapeutic potential in more complex wound environments.*

## 4 Experimental section

### 4.1 Materials

Chemical reagents and biological materials were used as received, without further purification. All experiments were conducted using deionized water and analytical-grade reagents.

*Chemical Reagents:* Zirconium chloride (ZrCl_4_), 2-aminoterephthalic acid (NH_2_–BDC), berberine hydrochloride (BBR), Ferulic acid (FA), N-(3-dimethylaminopropyl)-N′-ethylcarbodiimide hydrochloride (EDC·HCl), N-hydroxysuccinimide (NHS), 2,2-diphenyl-1-picrylhydrazyl **(**DPPH, D273092), and 2,2′-azino-bis(3-ethylbenzothiazoline-6-sulfonic acid (ABTS, A109612) were acquired from Aladdin in Shanghai, China. Resazurin (R7017) and C11-BODIPY_581_/_591_ (SML3717) were sourced from Sigma-Aldrich in the USA.

*Cell Biology Reagents:* Cell-based assay kits, including Cell Counting Kit-8 (CCK-8, CA1210), 2,7-dichlorodihydrofluorescein diacetate (DCFH-DA, D6470), and the 5,5,6,6-tetrachloro-1,1,3,3′-tetraethylbenzimidazolylcarbocyanine iodide (JC-1) mitochondrial membrane potential assay kit (M8650), were supplied by Solarbio (Beijing, China). Additionally, crystal violet staining solution (C0121) and a Live/Dead bacterial staining kit (C2030S) were obtained from Beyotime Biotechnology in Shanghai, China.

*Cell Lines:* The cell lines, RAW264.7 (RRID: CVCL_0493, CL-0190) and a normal primary human umbilical vein endothelial cell (HUVECs, RRID: CVCL_2959, CP–H082) were obtained from Procell Life Science & Technology Co., Ltd., Wuhan, China. Both cell lines were authenticated by short tandem repeat (STR) profiling and routinely tested to confirm the absence of mycoplasma contamination before use.

### 4.2 Methods

#### 4.2.1 Synthesis of UiO-66-NH_2_ and BBR + FA@UiO nanocomposites

The synthesis of UiO-66-NH_2_ was conducted using a modified solvothermal technique, as previously described [11]. In this procedure, equimolar amounts of ZrCl_4_ and 2-aminoterephthalic acid (NH_2_–BDC), each weighing 0.25 g, were dissolved in 50 mL of N, N-dimethylformamide (DMF). The solution was stirred for 30 min and subsequently heated to 120 °C for 24 h. The resulting solid product was then purified through multiple washings with DMF and ethanol, followed by vacuum drying at 60 °C.

To functionalize the material with FA, a conjugation reaction was employed. First, FA was activated by reacting it with EDC·HCl and NHS in ethanol for 2 h. The activated FA solution was then combined with UiO-66-NH_2_, and the mixture was stirred for 12 h to facilitate an amidation reaction between the carboxylic acid groups of FA and the amine groups on the MOF framework, yielding the FA-conjugated product (FA@UiO). This product was isolated by centrifugation, washed with ethanol, and dried.

Finally, the drug loading capacity (DLC) and the loading efficiency (LE) were achieved by dispersing the FA@UiO nanoparticles in an aqueous berberine (BBR) solution and stirring the mixture for 12 h at room temperature. The final nanocomposite, designated BBR + FA@UiO, was collected via centrifugation, washed to remove unloaded drug, and vacuum-dried. The amount of loaded drug was calculated as:

Loaded drug (mg) = Total drug input (mg) – Free drug in supernatant (mg)

DLC and EE were then calculated using the following formulas:

$$\text{DLC}\;\left(\text{wt}\%\right)=\frac{\text{Mass}\;\text{of}\;\text{loaded}\;\text{drug}\;\left(\text{mg}\right)}{\text{Mass}\;\text{of}\;\text{BBR}+\text{FA}@\text{UiO}\;\text{nanocomposite}\;\left(\text{mg}\right)}\times 100\;\%$$

$$\text{EE}\;\left(\%\right)=\frac{\text{Mass}\;\text{of}\;\text{drug}\;\text{initially}\;\text{added}\;\left(\text{mg}\right)}{\text{Mass}\;\text{of}\;\text{loaded}\;\text{drug}\;\left(\text{mg}\right)}\times 100\;\%$$

#### 4.2.2 Characterization of the BBR + FA@UiO nanocomposites

The nanocomposite's morphology and particle dimensions were characterized using a Zeiss Sigma 500 field-emission scanning electron microscope (FE-SEM). A Malvern Zetasizer Nano ZS instrument was used to measure hydrodynamic diameter and zeta potential. The crystalline structure was identified by X-ray diffraction (XRD) on a Bruker D8 Advance diffractometer with Cu Kα radiation. Chemical functional groups were analyzed by Fourier-transform infrared (FT-IR) spectroscopy using a Nicolet iS50 spectrometer. Optical properties were evaluated by collecting UV–vis spectra on a Shimadzu UV-2600 spectrophotometer, and surface elemental composition was determined via X-ray photoelectron spectroscopy (XPS) on a Thermo Fisher K-ALPHA system with Al Kα radiation.

#### 4.2.3 In vitro drug release behavior assay

The drug's pH- and reactive oxygen species (ROS)-dependent release profile was evaluated *in vitro*. Specifically, 5 mg of BBR + FA@UiO was suspended in 10 mL of phosphate-buffered saline (PBS) at varying pH levels (7.4, 6.5, and 5.5), with an additional condition of pH 5.5 containing 100 μM H_2_O_2_ to simulate a ROS environment. The suspensions were incubated at 37 °C under constant agitation. At predetermined intervals, 1 mL of the supernatant was extracted and replaced with an equal volume of fresh PBS to maintain sink conditions. The concentrations of released BBR and FA were quantified using UV–vis spectroscopy, measuring their absorbance at 345 nm and 320 nm, respectively. Cumulative drug release was determined by applying the measured concentrations to pre-established calibration curves, using a standard formula as follows:

$$\text{Released}\;\left(\%\right)=\left(\frac{2\times C_{t}}{2\times m_{0}}\right)\times 10$$

Where *C*_*t*_ represents the concentration at time *t*, and *m*_*0*_ is the initial mass of drugs loaded into the UiO-66-NH_2_ MOF.

#### 4.2.4 Antioxidant assays (DPPH• and ABTS•+)

The antioxidant potential of the BBR + FA@UiO nanocomposite was assessed via scavenging assays against DPPH• and ABTS•+ free radicals. In the DPPH• procedure, a 1 mL aliquot of a BBR + FA@UiO suspension (2.5–20 μg/mL) was combined with 1 mL of a 0.1 mM ethanolic DPPH• solution. This mixture was kept in darkness for 30 min before its absorbance was measured at 517 nm to determine the scavenging efficiency, as previously described [10].

Moreover, for the ABTS•+ assay, the radical cation was first generated by enabling a 7 mM ABTS solution to react with 2.45 mM potassium persulfate for 12 h in the dark. The resulting solution was then diluted to an absorbance of 0.70 (±0.02) at 734 nm. Subsequently, this working solution was incubated with the BBR + FA@UiO samples for 10 min, and the absorbance at 734 nm was recorded using a UV spectrophotometer. The antioxidant activity for both assays was calculated as a percentage using the formula:

$$\text{Antioxidant}\;\text{activity}\;\left(\%\right)==\left[1‐\frac{A_{s}}{A_{c}}\right]\times 10$$

where *A*_*c*_ is the absorbance of the control (without sample) and *A*_*s*_ is the absorbance of the sample measured at 517 nm or 734 nm, respectively.

#### 4.2.5 Scavenging of physiologically relevant ROS

The scavenging ability of BBR + FA@UiO was evaluated toward physiologically relevant ROS, including hydroxyl radicals (·OH) and hydrogen peroxide (H_2_O_2_).

Hydroxyl radical (• OH) scavenging assay: The OH scavenging activity was assessed using a Fenton reaction–based method. Briefly, hydroxyl radicals were generated by reacting Fe^2+^ with H_2_O_2_ in an aqueous system. BBR + FA@UiO at different concentrations (0, 2.5, 5, 10, and 20 μg/mL) was added to the reaction mixture, followed by incubation at room temperature for a fixed period. The remaining •OH was quantified using a colorimetric probe, and absorbance was recorded using a microplate reader. The •OH scavenging efficiency was calculated relative to a control group without BBR + FA@UiO.

Hydrogen peroxide (H_2_O_2_) scavenging assay: The H_2_O_2_ scavenging capacity of BBR + FA@UiO was determined by incubating different concentrations of the nanocomposite (0, 2.5, 5, 10, and 20 μg/mL) with a concentration of 2 mM H_2_O_2_ solution at 37 °C for 24h. After incubation, the residual H_2_O_2_ was quantified using a colorimetric assay based on the oxidation of a chromogenic substrate, and absorbance was measured with a microplate reader. The scavenging efficiency was calculated by comparing the absorbance values with those of the control group.

#### 4.2.6 Antibacterial and antibiofilm evaluation

##### 4.2.6.1 Bacterial culture and treatment

Antibacterial efficacy of BBR + FA@UiO was evaluated against two significant drug-resistant bacteria models, methicillin-resistant *Staphylococcus aureus* (MRSA, ATCC 43300), representing Gram-positive bacteria, and *Pseudomonas aeruginosa* (PA, ATCC 27853), representing Gram-negative bacteria. Following an overnight culture in Luria-Bertani (LB) broth at 37 °C with agitation (180 rpm), the bacterial suspensions were standardized to approximately 1 × 10^6^ colony-forming units (CFU) per mL. The experimental design comprised six distinct groups: an untreated Control, free BBR, free FA, UiO-66-NH_2_, a physical mixture of BBR + FA, and the BBR + FA@UiO nanocomposite. The treated group containing equivalent concentrations of BBR and FA (50 μg/mL) was co-incubated with the bacterial suspensions for 12 h at 37 °C. Subsequently, 10 μL aliquots from each group were serially diluted (from 10^−1^ to 10^−5^) and spread onto LB agar plates, which were incubated for 24 h at 37 °C. The number of colonies formed was enumerated, and the percentage of bacterial viability was determined by comparing the CFU count of each treatment group to that of the control.

##### 4.2.6.2 Live/dead bacterial staining

Bacterial membrane integrity was assessed with a Live/Dead Bacterial Viability Kit using DMAO and propidium iodide (PI). After a 12-h treatment, the bacterial suspensions were centrifuged at 5000 rpm for 5 min, then washed 3 times with PBS. The resulting pellets were resuspended and stained in the dark for 15 min with DMAO (5 μM) and PI (10 μM). After two additional PBS washes, the samples were visualized via fluorescence microscopy. In this assay, viable bacteria with intact membranes fluoresce green (live) from DMAO staining, whereas non-viable or membrane-damaged cells fluoresce red (dead) due to PI uptake.

##### 4.2.6.3 Biofilm formation and inhibition assay

The capability of the different composites to inhibit biofilm formation was assessed via a crystal violet staining assay. MRSA and PA suspensions, standardized to 1 × 10^6^ CFU/mL, were introduced into 24-well plates with LB broth and the different tested composite formulations (Control, BBR, FA, UiO-66-NH_2_, BBR + FA, or BBR + FA@UiO). Following a 24-h incubation at 37 °C, non-adherent cells were discarded, and the wells were rinsed with PBS. The remaining adherent biofilms were then fixed with paraformaldehyde, stained with 0. crystal violet, and subsequently washed. The dye bound to the biofilm was released using 3 acetic acid, and its absorbance was quantified at 595 nm. Biofilm biomass was expressed relative to the untreated control. The bacterial biofilm formation inhibition rate was calculated as follows:

$$Biofilm\;Inhibition\;Rate\;\left(\%\right)=\left[1‐\left(\frac{OD\_treated\;\mathrm{cells}}{OD\_control}\right)\right]\times 100\;\%$$

Where the optical density (OD) of treated cells represents the absorbance value at 595 nm for a well-treated with one of the composite formulations (BBR, FA, UiO-66-NH_2_, BBR + FA, or BBR + FA@UiO), and the OD control represents the absorbance value at 595 nm for the untreated control well.

#### 4.2.7 In vivo infected wound model experiments

Animal procedures were conducted in accordance with the national standards for laboratory animal care. They were approved by the Institutional Animal Care and Use Committee of Jiangsu Normal University (Approval No. [JSNU-IACUC-2025053]). The study used male ICR mice (6–8 weeks old, 25–30 g) that were acclimated for one week under controlled conditions (22 ± 2 °C, 55 ± 1 humidity, 12 h light/dark cycle) with unrestricted access to food and water. Following anesthesia using isoflurane, the dorsal area of each mouse was shaved and disinfected with 7 ethanol. A sterile biopsy punch was then used to create a 10 mm full-thickness circular wound on the mid-dorsal region, and any bleeding was controlled by applying gentle pressure with sterile gauze.

To model a bacterial infection, 50 μL of the MRSA suspension (1 × 10^8^ CFU/mL) was applied to each wound. The site was covered with a sterile transparent dressing for 12 h to facilitate bacterial colonization. Once the infection was established, the mice were randomly assigned to one of six treatment groups (n = 5 per group): Control (PBS), BBR, FA, UiO-66-NH_2_, a physical mixture of BBR + FA, and BBR + FA@UiO. Each treatment was prepared in PBS at 1 mg/mL, and 100 μL of the respective solution was administered topically to the wound every two days. After each application, the wound was covered with a fresh treatment, and the nanocomposite suspension in PBS adhered well, preventing loss of the formulation; thus, an overlying dressing was not required. This treatment concentration (1 mg/mL in PBS) was selected based on the *in vitro* antibactericidal efficacy of BBR + FA@UiO. This dose represents a conservative 2–4-fold elevation to ensure adequate drug exposure in the wound bed, accounting for potential dilution and biofilm barriers. Over the 11-day experimental time, body weight and wound condition were monitored at regular intervals (days 0, 3, 5, 7, 9, and 11). Wounds were photographed under standardized conditions, and the wound area was measured using ImageJ software. On day 11, the mice were euthanized, and wound tissue samples were collected for analysis. For bacterial load quantification, the tissues were homogenized in sterile PBS, serially diluted, plated on LB agar, and incubated at 37 °C for 24 h, after which colony-forming units (CFUs) were counted.

$$\text{Wound}\;\text{area}\;\left(\%\right)=\left[\frac{W_{t}}{W_{0}}\right]\times 100\;\%$$

Where *W*_*0*_ and *W*_*t*_ represent the initial and current wound areas, respectively.

#### 4.2.8 Histology and immunofluorescence of wound tissues

The experimental protocol was concluded after 11 days of treatment with the euthanasia of experimental mice. Harvested wound samples were immediately placed in paraformaldehyde for a 24-h fixation time. Following standard histological processing, the tissues were embedded in paraffin wax and serially sectioned at 5 μm. To assess morphological changes and collagen content during wound repair, histological staining was carried out using hematoxylin and eosin (H&E) and Masson's trichrome techniques.

Concurrently, immunofluorescence procedures were conducted. Tissue sections were incubated overnight at 4 °C with a primary antibody (dilution 1:200, Abcam) specific for CD31, VEGF, Nrf2, and HO-1. Detection was achieved using polymer-based, HRP-labeled anti-rabbit fluorescent secondary antibodies applied at 37 °C. Cellular nuclei were identified with DAPI counterstain. All fluorescent images were captured using fluorescence microscopy, and subsequent quantification of fluorescence signal intensity was performed using ImageJ.

#### 4.2.9 In vivo ELISA analysis of inflammatory cytokines in wound tissues

At the experimental endpoint, wound tissues from different treatment groups were harvested on day 11 and immediately weighed. The tissues were homogenized in ice-cold lysis buffer containing protease inhibitors, then centrifuged at 12,000 rpm for 15 min at 4 °C to collect the supernatants. The total protein concentration of each sample was determined using a BCA protein assay kit. The levels of pro-inflammatory cytokines, including tumor necrosis factor-α (TNF-α), interleukin-1β (IL-1β), and interleukin-6 (IL-6), in wound tissue homogenates were quantified using commercially available ELISA kits according to the manufacturers’ instructions. Cytokine concentrations were normalized to total protein content and expressed as pg/mg of protein.

#### 4.2.10 Western blot analysis

Proteins were isolated from wound tissue homogenates using RIPA lysis buffer enhanced with protease inhibitors. For immunoblotting, a consistent load of 30 μg of total protein per lane was separated by SDS-PAGE and subsequently transferred to a PVDF membrane. The membranes were blocked with bovine serum albumin (BSA) to prevent non-specific binding, then incubated with primary antibodies (1:1000, Cell Signaling Technology) overnight. The primary antibodies targeted a range of factors involved in oxidative stress, signaling, and angiogenesis (Nrf2, HO-1, NQO1, Keap1, β-catenin, TGF-β, Wnt, CD31, VEGF, Collagen I, TIMP-1, eNOS, HIF-1α, and Ang1). After washing, the blots were treated with appropriate HRP-conjugated secondary antibodies. Immunoreactive bands were revealed using an enhanced chemiluminescence (ECL) substrate (Bio-Rad), and band densities were quantified with Image Lab software.

#### 4.2.11 Statistical analysis

Statistical data were analyzed using IBM SPSS software (v21.0, IBM, USA). Data were presented as mean ± standard deviation (SD), and all figures were created using GraphPad Prism, version 8.3. To compare differences between groups, a one-way ANOVA followed by Tukey's post-hoc test was applied. statistical significance was defined as *∗p < 0.05, ∗∗p < 0.01, ∗∗∗p < 0.001*.

## CRediT authorship contribution statement

**Chen Chen:** Data curation, Investigation, Writing – original draft. **Na Zhang:** Data curation, Investigation. **Fructueux Modeste Amona:** Investigation, Validation, Writing – original draft. **Xiaolei Han:** Data curation, Investigation, Validation. **Qi Tang:** Data curation, Investigation, Validation. **Lanxin Geng:** Data curation, Investigation, Validation. **Guangfu Liao:** Conceptualization, Supervision, Writing – review & editing. **Jie Zhang:** Funding acquisition, Investigation, Writing – review & editing. **Tushuai Li:** Funding acquisition, Supervision, Writing – review & editing.

## Declaration of competing interest

The authors declare that they have no known competing financial interests or personal relationships that could have appeared to influence the work reported in this paper.

## Data Availability

Data will be made available on request.
