# Supplementary material for: Bridging traditional Chinese medicine theory with advanced MOFs delivery for synergistic drug-resistant infected-chronic wound therapy
Source: Mater Today Bio. 2026 Jan 30;37:102861. doi: 10.1016/j.mtbio.2026.102861 (PMC12890853; doi:10.1016/j.mtbio.2026.102861)
Supplement: Multimedia component 1 [file mmc1.docx]

Supporting Information

**Bridging traditional Chinese medicine theory with advanced MOFs delivery for synergistic drug-resistant infected-chronic wound therapy**

*Chen Chen^#^, Na Zhang^#^, Fructueux Modeste Amona^#^, Xiaolei Han, Qi Tang, Lanxin Geng, Guangfu Liao*, Jie Zhang*, Tushuai Li**

C. Chen

College of Hydraulic Engineering, Jiangsu Vocational Institute of Architectural Technology, Xuzhou, 221000, Jiangsu, China

N. Zhang

School of Food and Biological Engineering, Xuzhou University of Technology, Xuzhou 221018, Jiangsu, China

F. M. Amona

Institute of Cellular and Molecular Biology, School of Life Science, Jiangsu Normal University, Xuzhou, 221116, Jiangsu, China

X. Han, Q. Tang, L. Geng, J. Zhang, T. Li

School of Biology and Food Engineering, Suzhou University of Technology, Suzhou 215500, Jiangsu, China

E-mail: zhangj23@cslg.edu.cn and [litushuai@cslg.edu.cn](mailto:litushuai@cslg.edu.cn)

G. Liao

College of Materials Engineering, Fujian Agriculture and Forestry University, Fuzhou 350002, China

Email: liaogf@fafu.edu.cn

*^#^*C. Chen, N. Zhang, and F. M. Amona contributed equally to this work

**Supplementary Experimental Section**

**1. Methods**

**1.1. Colloidal Stability Evaluation by Dynamic Light Scattering (DLS)**

The colloidal stability of BBR+FA@UiO under biologically relevant conditions was evaluated by dynamic light scattering (DLS). BBR+FA@UiO was dispersed in H₂O, PBS, 0.9% NaCl, and DMEM supplemented with 10% fetal bovine serum (FBS) at a fixed concentration and stored at room temperature. At predetermined time points (0–7 days), the hydrodynamic diameter of the nanoparticles was measured using a DLS instrument.

**1.2. Cellular oxidative stress and mitochondrial function assays**

*1.2.1. Cell culture and oxidative stress model establishment*

RAW264.7 macrophages were cultured in DMEM supplemented with 10% fetal bovine serum (FBS) and 1% penicillin-streptomycin at 37 °C under a 5% CO₂ atmosphere. To model oxidative stress, cells were first exposed to 200 μM hydrogen peroxide (H₂O₂) for 4 hours to promote ROS generation. Subsequently, the cells were incubated for 12 hours with each of the different composite formulations (Control, BBR, FA, UiO-66-NH₂, BBR + FA, or BBR + FA@UiO).

*1.2.2. Intracellular ROS detection*

The quantification of intracellular ROS was conducted with the fluorescent indicator DCFH-DA (10 μM). Following the experimental treatments, the cell cultures were rinsed twice with phosphate-buffered saline (PBS) and subsequently loaded with the DCFH-DA working solution. This incubation was carried out for 30 minutes at 37°C under light-protected conditions. After removing unincorporated dye via additional PBS washes, fluorescence microscopy was used to visualize the cells. To quantify ROS production, the mean fluorescence intensity was calculated from at least 5 randomly selected fields of view using ImageJ.

*1.2.3. Mitochondrial membrane potential (ΔΨm) assay*

The mitochondrial membrane potential (ΔΨm) was assessed with the JC-1 assay kit according to the manufacturer's protocol. Treated cells were incubated with JC-1 dye (5 μg/mL) for 20 minutes at 37 °C, then washed. A fluorescence microscope was used to capture images, and the resulting ΔΨm was quantified as the red/green fluorescence ratio using ImageJ.

*1.2.4. Cell viability assay*

Cellular viability under oxidative stress conditions was assessed with a Cell Counting Kit-8. RAW264.7 cells were seeded in 96-well plates at a density of 1 × 10⁴ cells/well and treated with different composite formulations (Control, BBR, FA, UiO-66-NH₂, BBR + FA, or BBR + FA@UiO). After the treatment incubation, 10 µL of CCK-8 solution was added to each well, and the plates were incubated for an additional 2 hours at 37 °C. Absorbance was then recorded at 450 nm using a microplate reader. The resulting viability data were normalized to the data from the control group.

*1.2.5.* *Immunofluorescence staining of Nrf2 and Keap1*

Immunofluorescence staining was conducted on treated RAW264.7 cells to visualize protein localization. The cells were first fixed for 15 minutes with 4% paraformaldehyde and then permeabilized for 10 minutes with 0.1% Triton X-100. To prevent non-specific antibody binding, a 1-hour block with 5% bovine serum albumin (BSA) was performed. Subsequently, the cells were exposed to primary antibodies targeting Nrf2 and Keap1, both diluted 1:200, and incubated overnight at 4°C. The following day, samples were treated for one hour at room temperature with Alexa Fluor 488– and 594–conjugated secondary antibodies (1:500 dilution), with all steps involving fluorophores carried out in the dark. Nuclei were identified by counterstaining with DAPI (1 µg/mL) for 10 minutes. A fluorescence microscope was used to acquire images, and the fluorescence intensities of Nrf2 and Keap1 were quantified using ImageJ.

**1.3. Cell proliferation and migration assays (HUVECs)**

***1.3.1.*** *Cell culture and oxidative stress model establishment*

Human umbilical vein endothelial cells (HUVECs) were maintained in an endothelial cell growth medium at 37 °C and 5% CO_2_ as previously described (10). The culture medium was supplemented with 10% fetal bovine serum (FBS) and 1% penicillin-streptomycin. To model oxidative stress, the cells were first treated with 200 µM hydrogen peroxide (H_2_O_2_) for four hours. Following this induction time, the cells were exposed to each of the different composite formulations, including Control, BBR, FA, UiO-66-NH₂, BBR + FA, or BBR + FA@UiO, for a further 12 hours. In all relevant treatments, the concentrations of BBR and FA were standardized to 50 µg/mL.

***1.3.2.*** *Cell viability assay (CCK-8)*

The viability of HUVECs was evaluated using the Cell Counting Kit-8. Cells were first seeded into 96-well plates at a density of 1 × 10⁴ cells/well and left to attach overnight. Following the induction of oxidative injury and subsequent sample treatments, each well received 10 µL of the CCK-8 reagent. The plates were then incubated for 2 hours at 37°C, after which the absorbance at 450 nm was recorded on a microplate reader. Viability was calculated as a percentage relative to the untreated control cells.

***1.3.3.*** *Intracellular ROS measurement*

Intracellular ROS were detected with the fluorescent probe DCFH-DA. Following experimental treatments, HUVEC monolayers were rinsed with PBS and incubated with the DCFH-DA solution for 30 minutes at 37°C in the dark. After a subsequent PBS wash, fluorescence images were captured using a microscope. The relative fluorescence intensity, representing ROS levels, was quantified from the images using ImageJ software.

***1.3.4.*** *EdU cell proliferation assay*

Assessment of cellular proliferation was conducted using a 5-ethynyl-2’-deoxyuridine (EdU) incorporation assay with a commercial kit, following the provided protocol. In this procedure, treated HUVECs were first pulsed with a 10 μM working solution of EdU for 2 hours at 37°C. The cells were then fixed with 4% paraformaldehyde, permeabilized with 0.3% Triton X-100, and incubated with the Click-iT reaction cocktail for 30 minutes in the dark. Subsequently, a fluorescent dye was covalently attached to the incorporated EdU via a 30-minute Click-iT reaction in the dark. Finally, all nuclei were counterstained with DAPI. Quantification involved capturing fluorescent images and calculating the proliferation rate as the percentage of EdU-positive nuclei relative to the total nuclear count, as follows.

**Proliferation Rate (%)** $\text{= }\left[ \frac{\text{Number of EdU-positive Nuclei }}{\text{Total Number of Nuclei}} \right]\text{× 100 \%}$

**Where EdU-positive nuclei** are those that have incorporated the EdU dye, indicating new DNA synthesis, **the total Number of Nuclei** is the number of nuclei stained with DAPI, representing the total cell population.

**1.3.5.** *Cell migration assay (Transwell)*

Cell migration was assessed with a Transwell assay. In this procedure, HUVECs at a density of 1 × 10⁵ cells per well in a serum-free medium were placed into the upper chamber of the Transwell inserts, which featured 8 μm pores. The lower chamber was filled with 600 μL of a chemoattractant medium consisting of complete ECM supplemented with 10% FBS and the experimental compounds. Following a 24-hour incubation time at 37 °C, any cells that had not migrated were carefully scraped from the top of the membrane using a cotton swab. The cells that had traversed the membrane to the lower side were then fixed with 4% paraformaldehyde, stained with 0.1% crystal violet, and subsequently visualized and counted under a light microscope (10).

**1.4. Biosafety evaluation of BBR+FA@UiO**

*Hemolysis assay:* Red blood cells (RBC) were isolated from fresh mouse blood (0.5 mL) and centrifuged at 3500 rpm for 5 minutes, as previously described (6,9). Cells were puriﬁed by washing three times with sodium thiobarbital solution to prepare a 5% (v/v) erythrocyte suspension. The RBC suspension was serially diluted with BBR+FA@UiO (5, 10, 20, 40, 80 µg/mL) and gently shaken at 100 rpm. After 1 hour of incubation at 37°C, samples were centrifuged at 3500 rpm for 5 min, and the absorbance of the obtained supernatant was measured at 545 nm using a spectrophotometer. The negative and positive controls for the assay were PBS and deionized water.

The hemolysis ratio was calculated using the formula:

Hemolysis ratio (%) = $\frac{\mathrm{OD}_{\mathrm{sample}} - \mathrm{OD}_{\mathrm{PBS}}}{\mathrm{OD}_{\mathrm{water}} - \mathrm{OD}_{\mathrm{PBS}}}$ × 100%.

OD_sample_ represents the absorbance of erythrocytes exposed to different concentrations of BBR+FA@UiO, respectively.

**Supplementary figures**


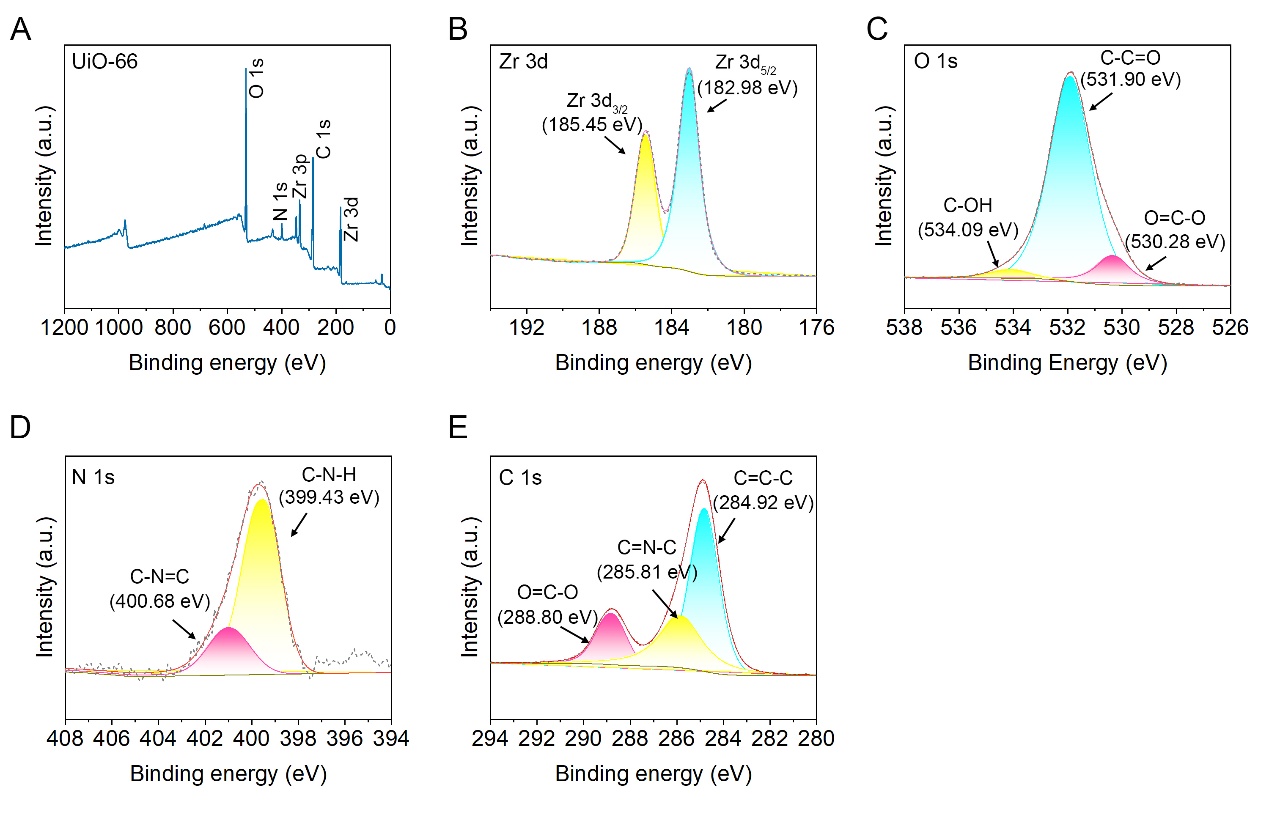


### Figure S1. XPS spectra of UiO-66-NH₂. (A) Survey spectrum of UiO-66-NH₂. (B) High-resolution Zr 3d spectrum. (C) High-resolution O 1s spectrum. (D) High-resolution N 1s spectrum. (E) High-resolution C 1s spectrum.

###
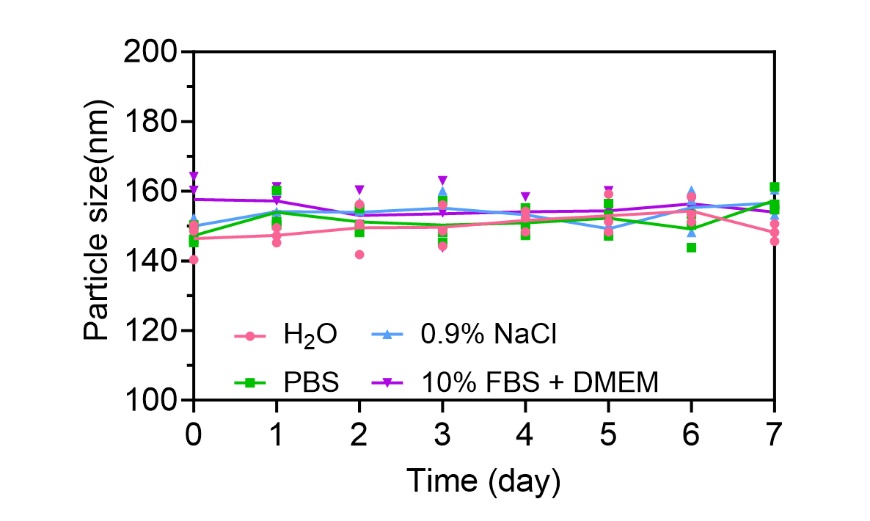


### Figure S2. Colloidal stability of BBR+FA@UiO under biologically relevant conditions. Dynamic light scattering (DLS) analysis showing the hydrodynamic diameter of BBR+FA@UiO after storage for up to 7 days in different media, including H₂O, PBS, 0.9% NaCl, and 10% FBS-supplemented DMEM. Data are presented as mean ± SD (n = 3).

###
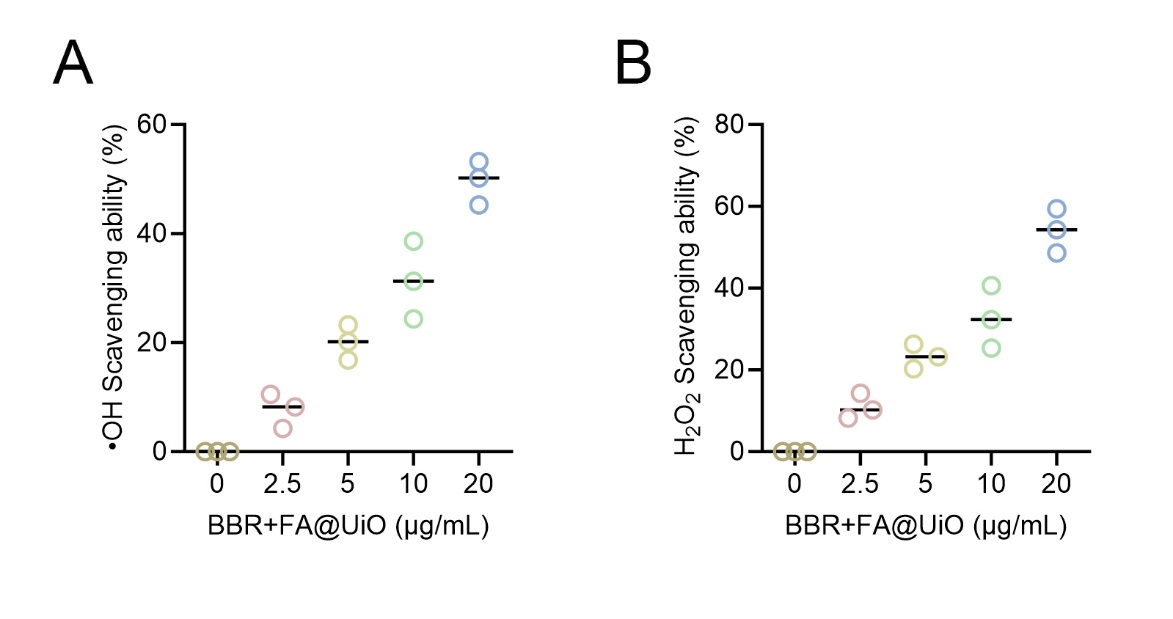


### Figure S3. Scavenging of physiologically relevant ROS by BBR+FA@UiO *in vitro*. Concentration-dependent scavenging ability of BBR+FA@UiO toward (A) hydroxyl radicals (·OH) and (B) hydrogen peroxide (H₂O₂), as evaluated by corresponding in vitro assays. Data are presented as mean ± SD (n = 3).


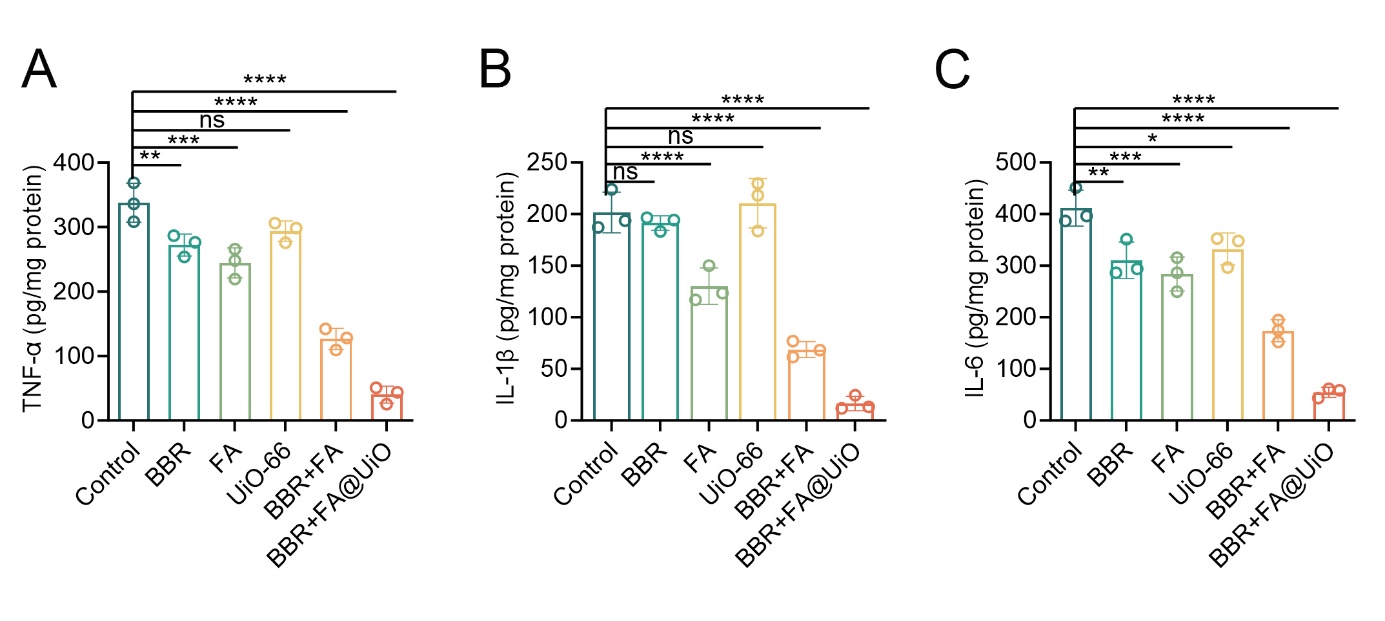


**Figure S4. *In vivo* inflammatory cytokine levels in wound tissues after treatment.** ELISA quantification of pro-inflammatory cytokines **(A) TNF-α**, **(B) IL-1β**, and **(C) IL-6** in wound tissue homogenates collected from different treatment groups. Data are presented as mean ± SD (n = 3). Statistical significance: ns, not significant; **p < 0.05, **p < 0.01, ***p < 0.001, ****p < 0.0001*.


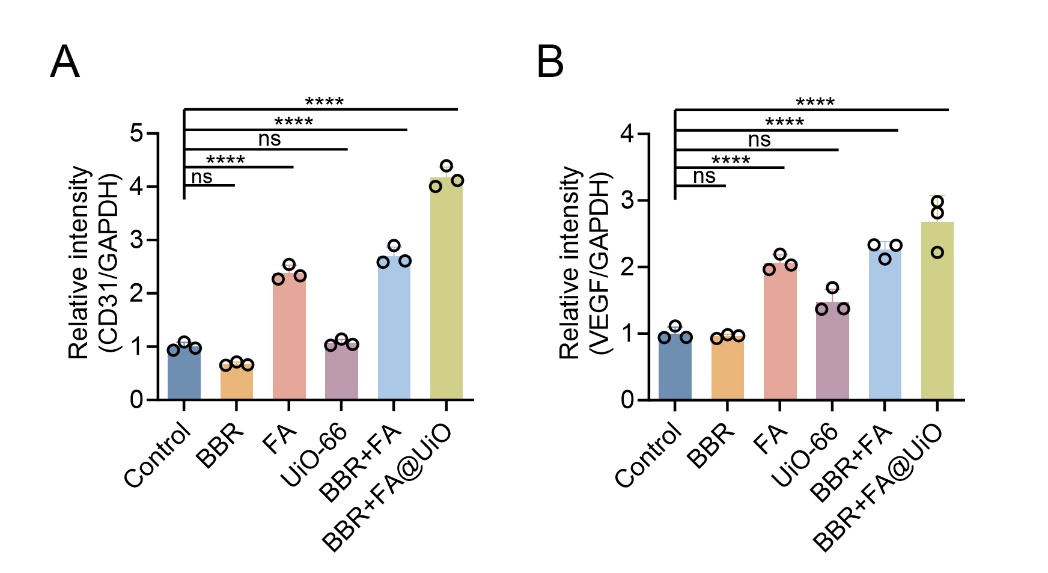


**Figure S5.** Western blot quantification of (A) CD31 and (B) VEGF expression levels shown in Fig. 7D. Protein expression was normalized to GAPDH, and data are presented as mean ± SD (n = 3). Statistical significance: ns, not significant; **p < 0.05, **p < 0.01, ***p < 0.001, ****p < 0.0001*.


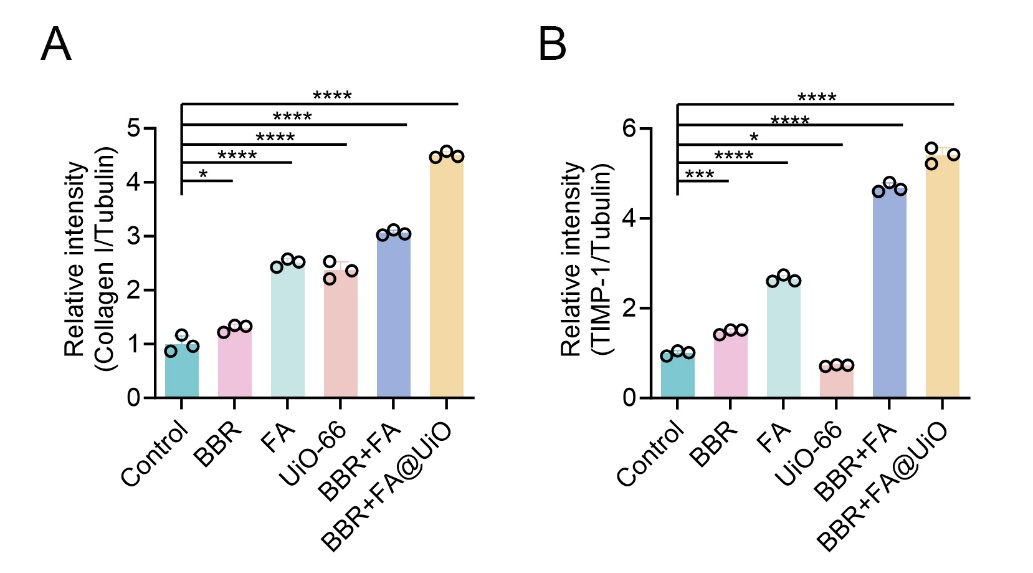


**Figure S6.** Western blot quantification of (A) Collagen and (B) TIMP-1 expression levels shown in Fig. 7E. Protein expression was normalized to GAPDH, and data are presented as mean ± SD (n = 3). Statistical significance: ns, not significant; **p < 0.05, **p < 0.01, ***p < 0.001, ****p < 0.0001*.


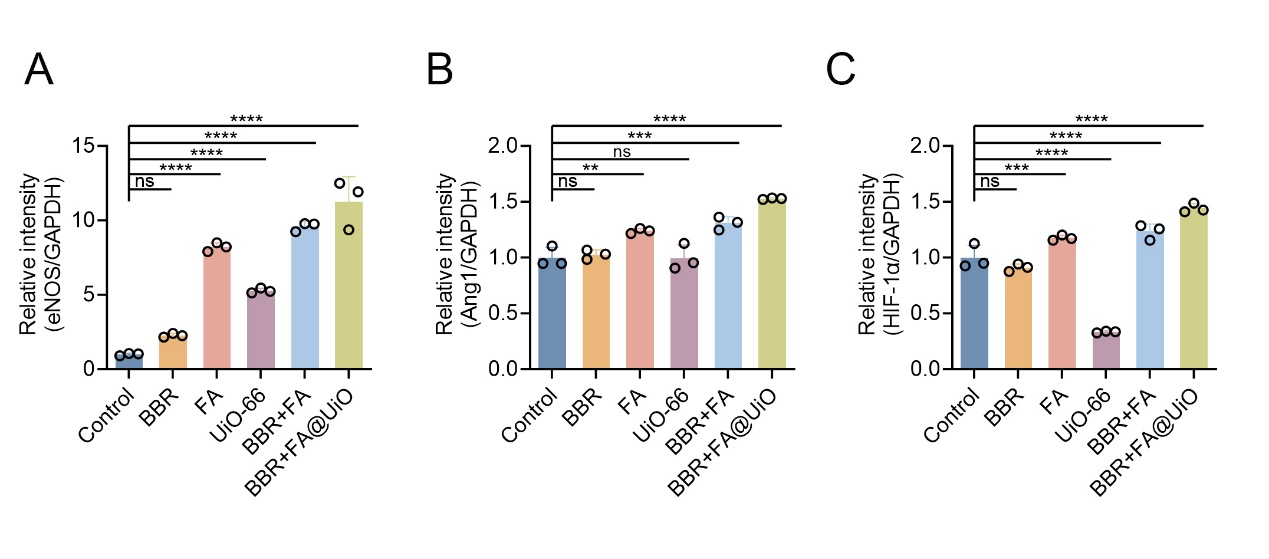


**Figure S7.** Western blot quantification of (A) Enos, (B) HIF-1α, and (C) Ang1 expression levels shown in Fig. 7F. Protein expression was normalized to GAPDH, and data are presented as mean ± SD (n = 3). Statistical significance: ns, not significant; **p < 0.05, **p < 0.01, ***p < 0.001, ****p < 0.0001*.


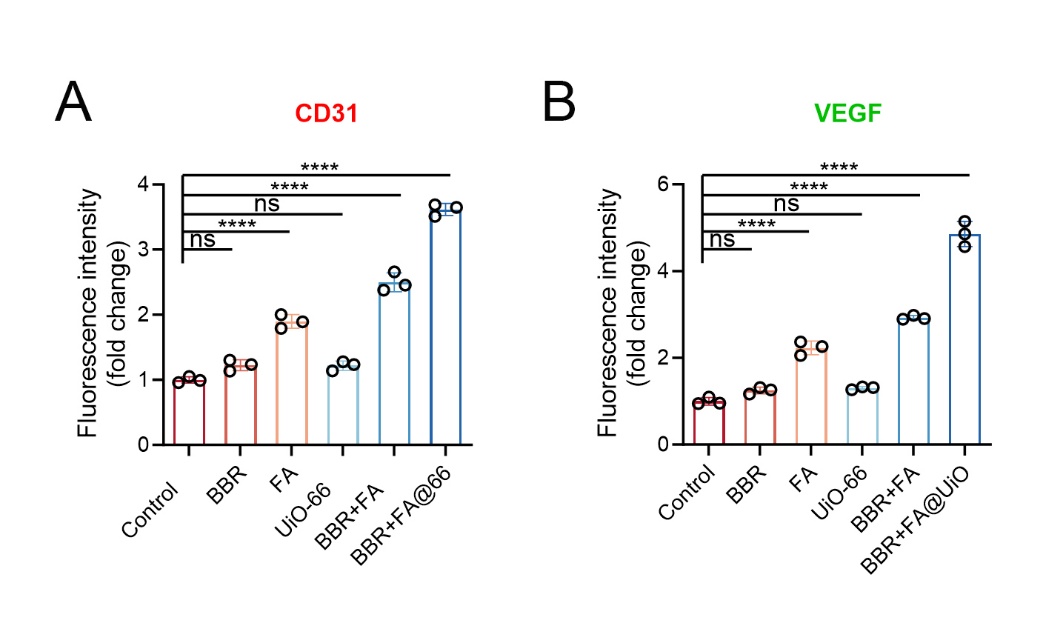


**Figure S8.** (A) Relative fluorescence intensity of CD31 (red) and (B) VEGF (green) in wound tissues from different treatment groups. Fluorescence intensity was quantified using ImageJ and normalized to the control group. Data are presented as mean ± SD (n = 3). Statistical significance: ns, not significant; **p < 0.05, **p < 0.01, ***p < 0.001, ****p < 0.0001*.


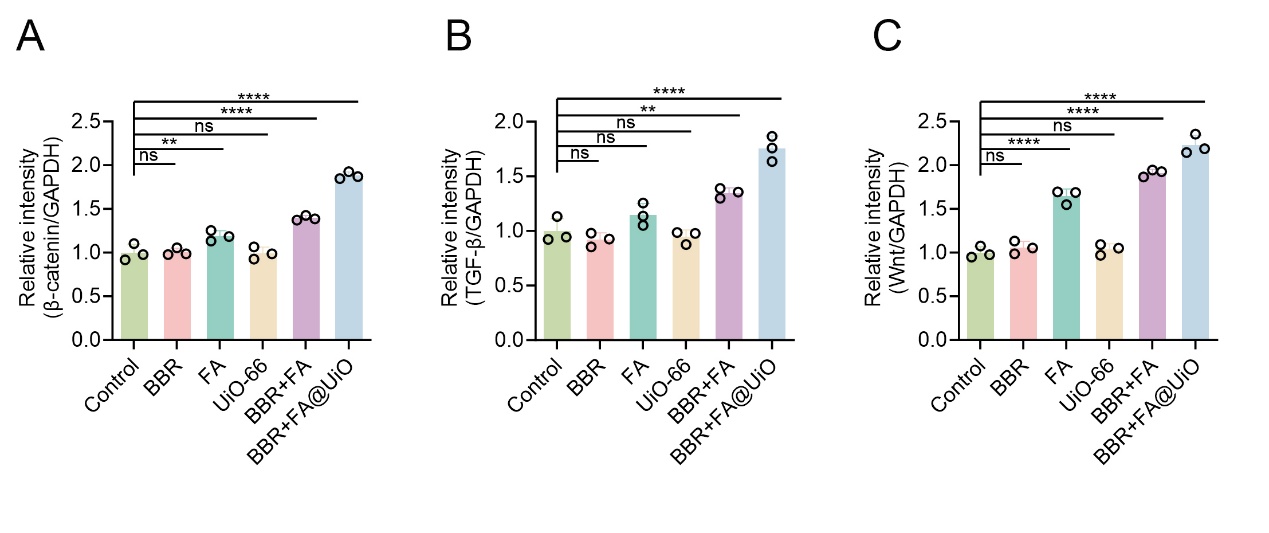


**Figure S9.** Western blot grayscale quantification of (A) β-catenin, (B) TGF-β, and (C) Wnt protein levels shown in Fig. 8B. Protein expression was normalized to GAPDH, and data are presented as mean ± SD (n = 3). Statistical significance: ns, not significant; **p < 0.05, **p < 0.01, ***p < 0.001, ****p < 0.0001*.


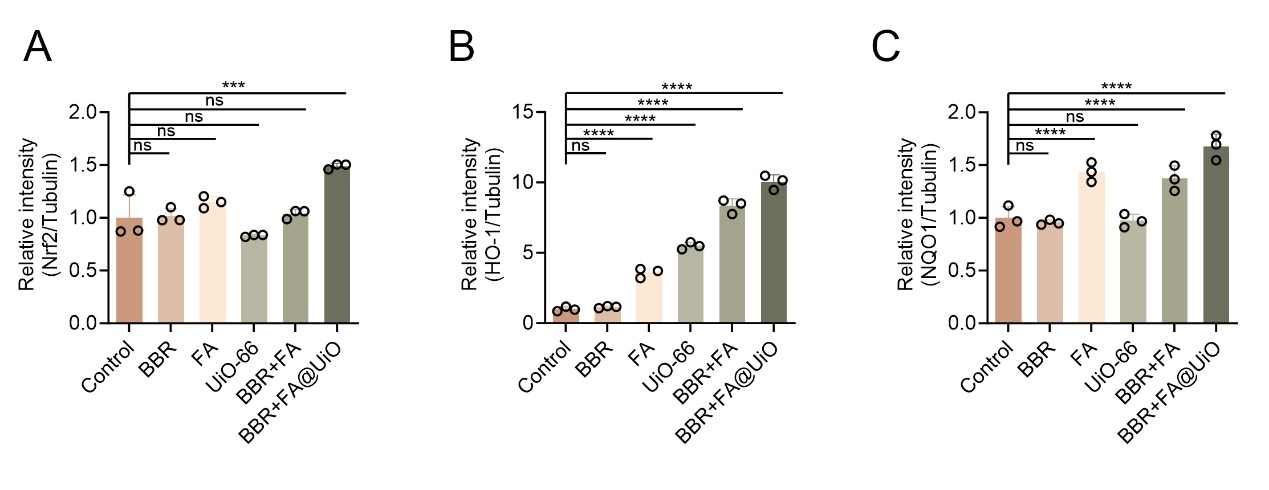


**Figure S10.** Western blot grayscale quantification of (A) Nrf2, (B) HO-1, and (C) NQO1 protein levels shown in Fig. 8B. Protein expression was normalized to GAPDH, and data are presented as mean ± SD (n = 3). Statistical significance: ns, not significant; **p < 0.05, **p < 0.01, ***p < 0.001, ****p < 0.0001*.
